# Mating Type Locus of Chinese Black Truffles Reveals Heterothallism and the Presence of Cryptic Species within the *T. indicum* Species Complex

**DOI:** 10.1371/journal.pone.0082353

**Published:** 2013-12-16

**Authors:** Beatrice Belfiori, Claudia Riccioni, Francesco Paolocci, Andrea Rubini

**Affiliations:** Institute of Biosciences and BioResources - Perugia Division, National Research Council, Perugia, Italy; Georg-August-University of Göttingen Institute of Microbiology & Genetics, Germany

## Abstract

*Tuber* spp. are filamentous ascomycetes which establish symbiosis with the roots of trees and shrub species. By virtue of this symbiosis they produce hypogeous ascocarps, known as truffles. Filamentous ascomycetes can reproduce by homothallism or heterothallism depending on the structure and organization of their mating type locus. The first mating type locus in a truffle species has been recently characterized in *Tuber melanosporum* and it has been shown that this fungus, endemic in Europe, is heterothallic. The availability of sequence information for *T. melanosporum* mating type genes is seminal to cloning their orthologs from other *Tuber* species and assessing their reproductive mode. Here we report on the organization of the mating type region in *T. indicum*, the black truffle species present in Asia, which is the closest relative to *T.* *melanosporum* and is characterized by an high level of morphological and genetic variability. The present study shows that *T. indicum* is also heterothallic. Examination of Asiatic black truffles belonging to different genetic classes, sorted according to the sequence polymorphism of the internal transcribed spacer rDNA region, has revealed sequence variations and rearrangements in both coding and non-coding regions of the mating type locus, to suggest the existence of cryptic species within the *T. indicum* complex. The presence of transposable elements within or linked to the mating type region suggests a role of these elements in generating the genotypic diversity present among *T. indicum* strains. Overall, comparative analyses of the mating type locus have thus allowed us to tackle taxonomical and phylogenetic issues within black truffles and make inferences about the evolution of *T. melanosporum-T. indicum* lineage. Our results are not only of fundamental but also of applied relevance as *T. indicum* produces edible fruit bodies that are imported also into Europe and thus may represent a biological threat for *T. melanosporum*.

## Introduction

Sexual reproduction, by virtue of its essential role in determining genetic variability and eliminating deleterious mutations, is at the origin of eukaryotic evolution. In fungi, sexual reproduction is controlled by genomic regions called mating type (*MAT*) loci [1], [2], [3]. The filamentous ascomycetes (Pezizomycotina) have a single *MAT* locus called *MAT1* [4], [5] with two master regulators of sexual reproduction: the *MAT1-1-1* and the *MAT1-2-1* genes encoding for an α-box and a high mobility group (HMG) domain protein, respectively [2]. Filamentous ascomycetes are haploid fungi that have two main sexual reproductive modes: homothallism and heterothallism. In homothallic species, both master *MAT* genes are present in the haploid nucleus of each strain. Thus, these fungi do not have distinctive mating types and are capable of haploid selfing as well as of crossing with any other strain [6]. Conversely, in heterothallic species, the two master *MAT* genes are located in different strains, thus haploid selfing is prevented and sexual reproduction can occur only between individuals of opposite mating type. The two alternative forms of the *MAT* locus in these fungi have been termed idiomorphs (*MAT1-1* and *MAT1-2*) rather than alleles because of their dissimilar sequences [7].

Within Pezizomycetes, the fungi belonging to the genus *Tuber* are organisms that establish symbiosis with the roots of many tree and some shrub species. By virtue of this symbiosis they produce hypogeous fruit bodies, known as truffles. Truffles produced by some species such as *Tuber melanosporum* Vittad. and *Tuber magnatum* Pico are edible and highly prized and appreciated by gourmets worldwide for their distinctive aroma. Thus, unearthing the reproductive biology of these species is not only of fundamental but also of applied relevance. The reproduction mode of truffles has been under debate for decades. Gaining direct evidence on their reproductive strategy is hampered by the difficulties of growing and the impossibility of mating these symbiotic fungal species under controlled conditions. Although mating type genes have been isolated from many members of the Pezizomycotina [8], their poor conservation across fungi made the identification of these genes in *Tuber* spp. by homologous cloning impossible [9]. Despite this, Bertault and colleagues [10] postulated the existence of a selfing reproductive system in *Tuber* spp. in order to account for the absence of heterozygotes observed when *T. melanosporum* ascocarps were screened with codominant simple sequence repeat (SSR) markers. However, few years later, an SSR-based screening aimed at investigating the genetic diversity within and among natural populations of *T. magnatum* showed that outcrossing events in this species were possible. As a matter of fact, two locus and multilocus linkage disequilibrium analyses documented the occurrence of an extensive genetic flow within and between populations [11]. Direct evidence of outcrossing was then gained when protocols were set to separately isolate and analyse the DNA contributed by the spores and the surrounding gleba from the same ascocarps. The evidence that the spores from a subsample of *T. magnatum* and *T. melanosporum* ascocarps displayed additional SSR alleles with respect to those showed by the surrounding gleba was interpreted to mean that these fungi can outcross and that gleba of their truffles is of uniparental origin [12], [13].

Finally, the sequencing of the *T. melanosporum* genome, strain MEL28, has revealed the presence of a single mating type (*MAT*) gene, namely the *MAT1-2-1* gene [14]. Because the second *MAT* gene, *MAT1-1-1*, has been cloned from a strain that lacked *MAT1-2-1* and the use of *MAT1-1-1* and *MAT1-2-1* specific primer pairs confirmed the presence of either *MAT1-1-1* or *MAT1-2-1* in the gleba of all *T. melanosporum* truffle investigated, it has been concluded that this fungus is heterothallic [14], [15]. An international patent covering the use for commercial purposes of the truffle *MAT* genes has been filed [16].

Different *Tuber* spp. produce edible black truffles. Because *T.* *melanosporum* has a distinctive taste, its commercial value is much higher than that of other species. Of these, the Asiatic species *T. indicum* Cooke and Massee has black ascocarps with morphological characteristics very similar to those of *T. melanosporum*. However, their flavor is inferior [17], [18]. Notably, the ectomycorrhizas formed by these two species are morphologically indistinguishable [19], [20]. Because the growing worldwide market for Chinese black truffles and their high level of similarity to *T. melanosporum,* they have been extensively studied over the last two decades. Indeed, black truffles of Asiatic origin may display many different morphotypes, leading to several authors claiming the presence of several other species such as *T. himalayense, T. sinense, T. pseudohimalayense, T. pseudoexcavatum* and *T. formosanum* in addition to *T. indicum* [21], [22], [23], [24], [25]. Extensive morphological and molecular analyses have confirmed the validity of only a few of these *taxa: T. pseudohimalayense* and *T. pseudoexacavatum* have been deemed to be conspecific, whereas *T. sinense, T. pseudohymalayense* and *T. formosanum* cannot be unambiguously distinguished from *T. indicum* [26]. With regard to the morphotypes belonging to *T. indicum sensu stricto*, extensive genetic variability was first revealed by studies based on RFLP analysis of the ITS region with the identification of at least three haplotypes referred to as *T. indicum*_A, *T. indicum*_B1 and *T. indicum*_B2 [27]. Subsequent studies based on ITS region and other phylogenetically informative genes such as 18S and 28S rDNA, β-tubulin and 1α-elongation factor have all confirmed the existence of two main clades within *T. indicum* [28], [29], [30]. However, due to their high morphological polymorphism, species boundaries within this group of fungi remain elusive [26], [31]. Nevertheless, molecular analyses have shown that *T. melanosporum* is closer to *T. indicum* than to the sympatric European black species such as *T. brumale* Vittad. [27], [30], [31], [32], [33], [34]. Based on phylogenetic analyses and current species distribution it has been also hypothesized that the Asiatic *T. indicum* and European *T. melanosporum* likely originated about 25–36 Ma ago by vicariance speciation [34]. The availability of sequence information for the *T. melanosporum MAT* genes is seminal to cloning their orthologs from Asiatic black truffles and assessing whether or not heterothallism is shared between closely related black truffle species. Even closely related ascomycetes may have different lifestyles and reproductive modes: for example, within the genera *Cochliobolus*, *Neurospora* and *Aspergillus,* both heterothallic and homothallic species have been described [35], [36], [37].

In addition, being considered rapidly evolving sequences [38], the *MAT* genes are ideal markers for gaining further insights into the taxonomy of Asiatic black truffles and the phylogeny of the *T. indicum* - *T.* *melanosporum* lineage.

The present study therefore sets out to: (i) isolate and characterize the *MAT* locus of *T. indicum* to ascertain the reproductive mode of this species; (ii) evaluate the extent of *MAT* polymorphism among different strains of *T. indicum* representative of the three genetic classes identified thus far and (iii) compare the sequences of the *MAT* genes and the structures of *MAT* idiomorphs of *T. indicum* and *T. melanosporum.* The discovery of the reproductive mode of *T. indicum* also has repercussions of an applicative nature, due to increasing interest in its cultivation in China [39] and a concern that this species, which is more competitive than *T. melanosporum* and potentially invasive, may represent a threat for *T. melanosporum* in Europe [27], [40], [41].

## Materials and Methods

### Fungal Samples and Morphological Analyses

A set of Asian black truffles imported into Italy and in France (Table S1) was analyzed for the morphology of ascocarps and ascospores as previously reported [18], [21], [26]. Their ascospores and in particular their sporal ornamentations were examined and photographed using a light microscope (Zeiss Axiophot) and a scanning electron microscope (SEM, Philips XL30). For light microscope analysis the spores were mounted in lactic acid; for SEM examination the ascospores, collected from ripened ascocarps, were washed in distilled water and preserved in 95% ethanol. A small drop containing the spores was placed on aluminum stubs covered with double-sided adhesive tape, dried at 25°C and coated with Gold-Palladium using an E1500 SEM Coating Unit (Polaron Equipment Ltd, Watford, UK). Lyophilized specimens of truffles used in this study are conserved at the CNR, Institute of Biosciences and BioResources, Perugia Division.

### DNA Isolation, PCR Amplification and Sequencing

Genomic DNA was isolated from ascocarps as described previously [12]. PCR amplification of the ITS region was performed using ITS1 and ITS4 primers [42]. The RFLP analysis of ITS amplicons was performed using the *Rsa*I endonuclease in accordance with the procedure of Paolocci et al. [27].

*T. indicum* α- and HMG-box fragments were amplified by PCR using the *T. melanosporum* primer pairs p1/p2 and p19/p20 and the cycling conditions reported in Rubini et al. [15] with the following modifications: the annealing temperature was decreased to 50°C and the number of cycles to 25.

The PCR primers used for cloning the *T. indicum MAT* genes and idiomorphs were designed with the help of PerlPrimer v. 1.1.16 software [43] and are reported in Table S2. PCR amplification of the mating type genes was performed in a 50 µl mixture containing 2.5 mM MgCl_2_, 0.2 mM of each dNTPs, 10 nM of each primer, 1 U of EuroTaq DNA polymerase (Euroclone, Milan, Italy) and 20 ng of DNA. PCR reactions were performed in a 9700 PCR system (Life Technologies, Carlsbad, CA, USA) using the following thermal profile: 2 min and 30 s at 94°C followed by 25–30 cycles of denaturation at 94°C for 30 s, annealing at 60°C for 30 s and extension at 72°C for 30–60 s depending on the length of the amplicons, and a final extension of 7 min at 72°C.

Long distance PCR amplification of *MAT1-1* and *MAT1-2* fragments was performed with LA-Taq DNA polymerase (Takara Bio Inc., Otsu, Japan) using a 2-step thermal profile: 30 s at 94°C followed by 25–30 cycles of denaturation at 94°C for 30 s and annealing/extension at 68°C for 10–15 min depending on the length of the amplicons, and a final extension of 7 min at 72°C.

To amplify both idiomorphs from a single ascocarp, the long-distance PCR protocol described above was modified by increasing the number of PCR cycles to 50. Sequencing of the PCR products was performed using the Big Dye sequencing kit V. 3.1 (Life Technologies, Carlsbad, CA, USA) in accordance with the manufacturer's protocol either on amplicons purified using a JetQuick PCR purification kit (Genomed, GmbH, Löhne, Germany) or on amplicons previously cloned in *E. coli* strain JM83 using standard protocols. Sequence visualization, editing and assembly were performed using FinchTV v. 1.3.1 (Geospiza, Inc., Seattle, WA, USA; http://www.geospiza.com) and Geneious v. 4.8.5 (Biomatters Ltd, Auckland, New Zealand; http://www.geneious.com). Gene prediction analyses were performed using FGENESH (http://linux1.softberry.com/berry.phtml).

### RNA Isolation and Reverse-transcription Polymerase Chain Reaction Analysis

Total RNA was isolated from *MAT1-1* and *MAT1-2* ascocarps stored at −80°C following Rubini et al. [15]. To confirm the structure of *MAT* genes, 5 µg of total RNA was reverse-transcribed using the SuperScript® III Reverse Transcriptase enzyme and 200 ng of random primers (Life Technologies, Carlsbad, CA, USA) according to the supplier’s instructions. First-strand cDNA was then amplified according to the PCR protocol reported above, using the primer pairs i11–i12 and i5–i13 for *MAT1-1-1* and *MAT1-2-1*, respectively (Table S2, Figure S4).

## Results

### Morphological and Molecular Characterization of *T.* *indicum* Samples

**Figure 1:**
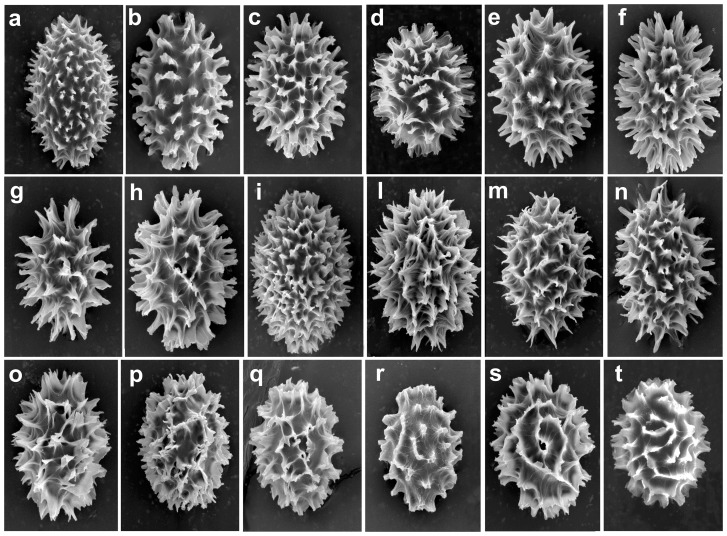
SEM images of *T. indicum* ascospores showing details of the ornamentation. a: Ti_C4; b: Ti_C80; c: Ti_C80; d: Ti_C55; e: Ti_C47; f : Ti_C55; g: Ti_C29; h: Ti_C55; i: Ti_C36; l: Ti_C36; m: Ti_C36; n: Ti_C15; o: Ti_C29; p: Ti_C18; q: Ti_CU3; r: Ti_C55; s: Ti_C47; t: Ti_C57.

Given the high genetic variability of Chinese black truffles, a set of 115 ascocarps all displaying the *T. indicum* morphotypes was analyzed for the polymorphism of the ITS rDNA region. The PCR amplification of the ITS region with universal ITS1/ITS4 primers produced an amplicon of approximately 600 bp in all samples. The ITS/RFLP analysis with *Rsa*I endonuclease allowed the classification of all the ascocarps into the 3 genetic classes (A, B1, B2) previously identified by Paolocci et al. [27] (Table S1). In order to evaluate putative morphological differences between truffles belonging to the different RFLP classes, a detailed analysis of ascospore morphology was performed with scanning electron microscope (SEM, Figure 1) and light microscope (Figures S1, S2 and S3). SEM analysis showed a highly variable sporal ornamentation ranging from the aculeate type with free small or large spines (Figure 1a–b) to the aculeate-reticulate type where the spines were more or less enlarged and connected at the base (e.g. Figure 1c–o) or even completely fused to give an irregular grid with closed or open meshes (Figure 1p–t). Examination of approximately fifteen truffles per RFLP class showed that spores with similar morphology were present in all of the truffle samples irrespective of RFLP class, making clear differentiation difficult. However ascocarps belonging to *T. indicum* RFLP class A displayed spores with free spines (Figure S1) more frequently than the samples belonging to the other two classes (Figures S2 and S3).

A subsample of eight *T. indicum* ascocarps for each of the three ITS classes was then used for the analysis of the mating type (*MAT*) genes and idiomorphs (Table 1).

**Table 1:** List of selected ascocarps analyzed for the mating type.

Sample	ITS/RFLP pattern	Mating type
Ti_C37	A	1-1
Ti_CF10	A	1-2
Ti_C69	A	1-1
Ti_CF11	A	1-2
Ti_C80	A	1-1
Ti_CF4	A	1-2
Ti_D5	A	1-1
Ti_D9	A	1-2
Ti_LI2	B1	1-1
Ti_U983	B1	1-2
Ti_RIBC	B1	1-2
Ti_C20	B1	1-1
Ti_D15	B1	1-1
Ti_D54	B1	1-1
Ti_F7	B1	1-2
Ti_F3	B1	1-2
Ti_C61	B2	1-1
Ti_U986	B2	1-2
Ti_CF2	B2	1-1
Ti_LI8	B2	1-2
Ti_CF7	B2	1-1
Ti_C8	B2	1-2
Ti_C18	B2	1-2
Ti_C38	B2	1-2

### Identification of *T. indicum MAT* Genes

The primer pairs p1/p2 and p19/p20 previously designed on the most conserved regions, namely the HMG-box and α-box domains, of *T. melanosporum MAT1-2-1* and *MAT1-1-1* genes, respectively [15], were used to amplify, under low-stringent PCR conditions (see M&M), orthologs of the *MAT* genes from the 24 selected *T. indicum* samples. As the DNA isolated from each single fruit body was mainly contributed by the gleba, and to a far lesser extent by the spores [15], the number of the PCR cycles for this screening was kept low (less than 30 cycles) to allow the preferential amplification of solely DNA isolated from the gleba. Using this approach, the primer pair p1/p2 produced a single amplicon of approximately 500 bp from 13 out of the 24 *T. indicum* ascocarps analyzed. The remaining 11 ascocarps yielded an amplicon of about 400 bp with the primer pair p19/p20 only. In every ITS class investigated, the presence of truffles whose gleba was made either by strains harboring the *MAT1-1-1* or the *MAT1-2-1* gene was balanced (Table 1).

The PCR fragments from three ascocarps that produced either the *MAT1-2-1* specific (Ti_U983, Ti_U986 and Ti_CF10) or the *MAT1-1-1* specific (Ti_C61, Ti_LI2, and Ti_C37) amplicon only were cloned and sequenced. Sequence analysis showed that these PCR fragments shared high similarity with HMG-box and α-box sequences of *T. melanosporum* respectively (data not shown). The finding that only one of the two *MAT* genes was amplified from all of the samples processed was interpreted to mean that, regardless of the morphotypes, *T. indicum* was heterothallic.

### Amplification of the 5′ and 3′ Ends of *T. indicum MAT* Idiomorphs

On the HMG-box and α-box sequences cloned as reported above, two *MAT1-2-1* primers, named i5 and i6, and two *MAT1-1-1* primers, named i3 and i4, were designed, respectively. For each *MAT* gene these primers were designed with in opposite orientations and to be combined with the primers i1 and i2 designed on the *T. melanosporum* 5′ and 3′ regions flanking the *MAT* locus and conserved between the strains of different mating types (Table S2, Figure S4). The primer pairs i1/i4 and i2/i3 produced fragments of about 1.4 Kbp and 8 Kbp, respectively, from all of the three *T. indicum* samples that produced the *MAT1-1-1* amplicon when amplified with the primer pair p19/p20 (Ti_LI2, Ti_C61 and Ti_C37). With regard to the samples that gave rise to the *MAT1-2-1* amplicon when amplified with the p1/p2 pair, two (Ti_U986 and Ti_CF10) yielded a fragment of about 8 Kbp and one (Ti_U983) a fragment of 11 Kbp with the primer pair i1/i6. The same samples produced a fragment of 3.5 Kbp (Ti_U983 and Ti_U986) and a fragment of 6.5 Kbp (Ti_CF10) with the primers i2/i5 (Figure S4).

These PCR-based approaches showed that the *MAT1-2-1* and *MAT1-1-1* genes had a similar orientation within the *MAT* loci of both *T. indicum* and *T. melanosporum.*

### Isolation of *T. indicum MAT* Idiomorphs

The 5′ and 3′ ends of the *T. indicum MAT* amplicons obtained as reported above were sequenced and aligned. The primers i8 and i9 were then designed on the 3′ ends of the *MAT1-2* and *MAT1-1* amplicons, respectively, whereas the primers i7 and i10 were designed on the 5′ and 3′ flanking regions shared between *MAT1-2* and *MAT1-1* idiomorphs, respectively (Table S2, Figure S4). Different primer combinations were finally used to amplify the complete *T. indicum* regions spanning the *MAT1-1* and *MAT1-2* idiomorphs from the *T. indicum* samples Ti_U983, Ti_U986 and Ti_CF10, selected on the basis of their different ITS/RFLP patterns (Table 1 and Figure S4). To reach this goal the number of PCR cycles was increased to 50 to allow the amplification from each *T. indicum* fruit body of the DNA contributed also by the spores.

Using this cycling condition and the primer pair i7/i10, two fragments of approximately 9 and 14 Kbp from ascocarps Ti_U983 and Ti_CF10, and 9 and 11 Kbp from ascocarp Ti_U986 were amplified. The intensity of the two bands produced *per* sample was different since the DNA contributed by the gleba always yielded the more intense band, as expected (Figure S5).

**Figure 2:**
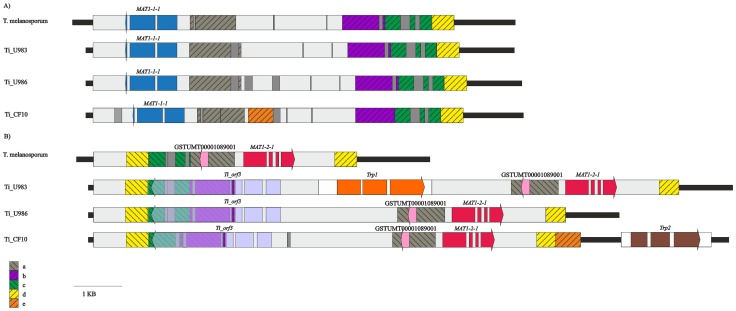
Schematic representation of the genomic regions containing the *MAT1-1* (A) and *MAT1-2* (B) idiomorphs of *T. melanosporum*, and *T. indicum* Ti_U983, Ti_U986 and Ti_CF10 samples, respectively. The grey boxes indicate the idiomorphic regions, black lines indicate the common flanking regions. The identified ORFs are indicated by blue (*MAT1-1-1*), red (*MAT1-2-1*), light blue (*Ti_orf3*), pink (gene model GSTUMT00001089001), orange (*Trp1*) and brown (*Trp2*) arrowed boxes, respectively. The white boxes indicate the putative TEs. The grey boxes indicate sequence insertions. The grey (a), purple (b), green (c), yellow (d) and orange (e) hatched boxes represent regions sharing sequence similarities between idiomorphs. The grid pattern indicates the opposite orientation of similar sequences.

Finally, by using the primers i8 or i9, specific to the idiomorphs *MAT1-2* and *MAT1-1*, respectively, in combination with the forward primer i7 common to both, the two *MAT* regions were separately amplified from each of the three *T. indicum* selected samples (Figure S4, Figure 2). The six amplicons obtained were sequenced and deposited in GenBank under accession n. KF318361–KF318366.

### Structure of *T. indicum MAT* Idiomorphs and Comparison with *T. melanosporum*

Sequence alignment, BLAST and gene prediction analyses using FGENESH were performed to evaluate the length and the structure of *MAT1-1* and *MAT1-2* idiomorphs and identify the putative coding regions therein. Figure 2 shows the organization of the *MAT* idiomorphs from *T. melanosporum* and the three *T. indicum* analyzed herein. The length of each idiomorphic region varied from 9.7 to 12.1 Kbp for the *MAT1-2* and from 7.6 to 7.7 Kbp for the *MAT1-1*. Within each sample the two *T. indicum* idiomorphs were not completely different as they shared sequences arranged both in the same (Figure 2B, orange (e) hatched box) or in inverse orientation (Figure 2, grey (a), purple (b), green (c), and yellow (d) hatched boxes). These regions were also shared with *T. melanosporum*, with the exception of the region “e” which was present only in the two idiomorphs of Ti_CF10, and region “b” which was absent from the *T. melanosporum MAT1-2* idiomorph.

### Structure of *MAT1-1* Idiomorph

The *MAT1-1* idiomorph was slightly longer in *T. indicum* (7,583 bp, 7,741 bp and 7,666 bp in Ti_U983, Ti_U986 and Ti_CF10, respectively) than in *T. melanosporum* (7,430 bp). The structure of *MAT1-1* idiomorph was very similar among the three *T. indicum* samples and *T. melanosporum.* The major difference resided in a region of about 750 bp, conserved in Ti_U983, Ti_U986 and *T. melanosporum*, which was replaced in Ti_CF10 by a completely unrelated sequence, “e” (Figure 2A, orange hatched box). The “e” sequence was also present at the 3′ end of the *MAT1-2* idiomorph in the same sample (Figure 2B, orange hatched box). A BlastN search identified several sequences similar to the region “e” of Ti_CF10 (about 85% identity) dispersed throughout the *T. melanosporum* genome and annotated as transposable elements (TEs) in the *T. melanosporum* database (http://mycor.nancy.inra.fr/IMGC/TuberGenome/blast.php). However, neither BlastN nor BlastX searches against the Genbank database revealed any significant match for this query. Moreover, the sequences of the *MAT1-1* of *T. indicum* differed from each other and from that of *T. melanosporum* for the presence of several indels ranging from a few bp to approximately 160 bp. All of these insertions are indicated by the grey boxes in Figure 2.

Gene prediction analysis of *MAT1-1* revealed the presence of a single gene (Figure 2A, blue arrowed box) exhibiting high similarity (97% sequence identity) with *T. melanosporum MAT1-1-1*. RT-PCR analysis of RNA isolated from Ti_U983, Ti_U986 and Ti_CF10 fruit bodies confirmed the presence for *MAT1-1-1* gene of a single transcript deriving from three exons. On the contrary in *T. melanosporum* two alternative *MAT1-1-1* transcripts were detected [15].

**Figure 3:**
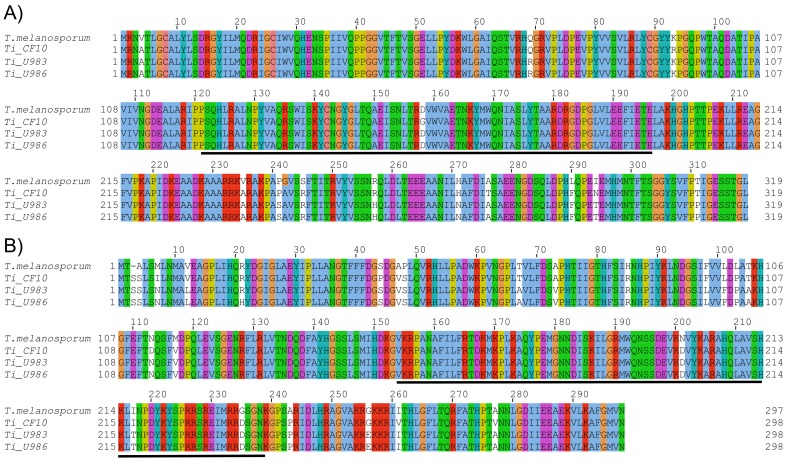
Amino acid alignment of *T. indicum* and *T. melanosporum MAT1-1-1* (A) and *MAT1-2-1* (B) deduced proteins. The HMG-box and α-box regions are underlined. The default ClustalX color code is used.

The *MAT1-1-1* gene of Ti_U983 differed from Ti_U986 for two nucleotides only, whereas Ti_CF10 differed from Ti_U983 and Ti_U986 for 28 and 30 nucleotides, respectively. The *T. indicum MAT1-1-1* sequences differed from that of *T. melanosporum* for 26, 28 and 27 nucleotides in Ti_U983, Ti_U986 and Ti_CF10, respectively (Table S3, Figure S6). The deduced MAT1-1-1 encoded protein is 319 amino acids long in each of the three *T. indicum* strains. Ti_U983 and Ti_U986 shared identical protein sequences, whereas Ti_CF10 contains eight amino acid changes compared with the other two strains. The MAT1-1-1 proteins of Ti_U986 and Ti_U983 differed from *T. melanosporum* MAT1-1-1 for eleven amino acids, whereas for Ti_CF10 it differed for eight amino acids only (Figure 3A).

### Structure of *MAT1-2* Idiomorph

The *T. indicum MAT1-2* idiomorph was 9,783 bp, 10,096 bp and 12,131 bp long in Ti_U986, Ti_CF10 and Ti_U983, respectively. Overall, this idiomorph was longer in *T. indicum* than in *T. melanosporum* (5,550 bp long), due to a large insertion, by about 4,300 bp in Ti_U986 and Ti_CF10 and about 6,650 bp in Ti_U983, between the “c” and “a” regions (Figure 2B, green and grey hatched boxes).

In contrast to *MAT1-1,* in which a single ORF was identified, three ORFs in Ti_U986 and Ti_CF10 and four ORFs in Ti_U983 were predicted within this idiomorph (Figure 2B). Among these ORFs, RT-PCR analysis confirmed the presence of the *MAT1-2-1* gene: this gene exhibited high similarity (94% sequence identity) and the same exon-intron structure as its ortholog from *T. melanosporum* (Figures 2B and S7). More specifically the *MAT1-2-1* gene of Ti_U983 differed from Ti_U986 for one nucleotide only, whereas Ti_CF10 differed from Ti_U983 and Ti_U986 for 38 and 39 nucleotides, respectively. The *T. indicum MAT1-2-1* sequences differed from that of *T. melanosporum* for 46, 48 and 49 nucleotides in Ti_CF10, Ti_U983 and Ti_U986, respectively (Table S3, Figure S7). *T. indicum MAT1-2-1* gene encoded a hypothetical protein of 298 amino acids with Ti_U983 differing from Ti_U986 for a single amino acid only. Conversely, Ti_CF10 differed for thirteen and fourteen amino acids from Ti_U983 and Ti_U986, respectively. In all, the *T. indicum* MAT1-2-1 deduced proteins coded by Ti_U986, Ti_U983 and Ti_CF10 differed from that of *T. melanosporum* for 19, 18 and 14 aa, respectively and for an insertion of a single serine in the N-terminal region (Figure 3B).

A second, short ORF was detected within the “a” region (Figure 2B, pink arrowed box) in all *T. indicum* samples. This ORF was similar to GSTUMT00001089001 previously identified within the same region of the *T. melanosporum MAT1-2* idiomorph [15] but was missing within the corresponding region exhibited by the *MAT1-1* idiomorphs of both *T. indicum* and *T. melanosporum*.

**Figure 4:**
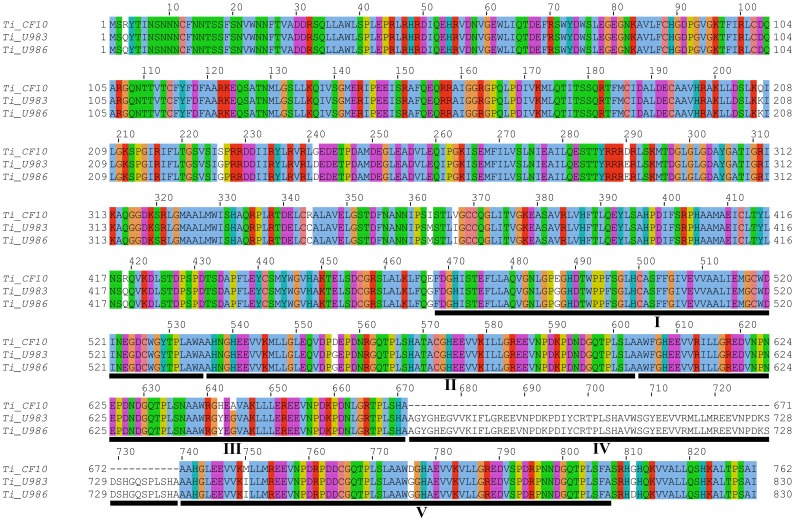
Amino acid alignment of *T. indicum Ti_orf3* encoded hypothetical protein. The ANK repeats are underlined.

The third putative gene identified in each *T. indicum MAT1-2* was named *Ti_orf3* and consisted of 4 exons in an inverse orientation with respect to the *MAT1-2-1* gene (Figure 2B). The *Ti_orf3* gene coded for a hypothetical protein of 830 aa in Ti_U986 and Ti_U983 and 762 aa in Ti_CF10 (Figure 4). BlastP analysis against the GenBank database showed high similarity (E-value <8e −45) with uncharacterized Ankyrin proteins of ascomycetes (i.e. XP_002487679, XP_002382023, XP_002144344). Searches against the Conserved Domains Database (CCD) at NCBI revealed the presence of conserved ankyrin domains (ANK) at the C-termini of the predicted amino acid sequence. Amino acid alignment showed that this hypothetical protein differed between the *T. indicum* samples for the number of ANK repeat units at their C-terminal region, being five in Ti_U986 and Ti_U983 and 4 in Ti_CF10 (Figure 4). The 4^th^ exon of *Ti_orf3* gene in the *MAT1-2* idiomorph spans the “b” and “c” regions in all *T. indicum* samples (Figure 2B, purple and green hatched boxes, respectively). Homologous regions, with about 85% nucleotide identity, were also present in an inverse orientation in the *MAT1-1* idiomorphs of both *T. indicum* and *T. melanosporum*. However, the absence of the first three exons along with the presence of several stop codons in the hypothetical 4^th^ exon made the presence of a functional *Ti*_*orf3* gene within the *MAT1-1* idiomorphs unlikely.

A fourth ORF was identified in the *MAT1-2* idiomorph of *T. indicum* Ti_U983 only. This was longer with respect to the others for the presence of a 2,351 bp long insertion (Figure 2B, orange box).

### Characterization of Transposable Elements in the *MAT* Locus of *T. indicum*

The 2,351 bp long insertion displayed by the Ti_U983 *MAT1-2* idiomorph revealed the presence of a TE which was named Ti_tr1. Gene prediction analysis and BlastX searches against the GenBank database in fact made it possible to identify a putative 1,819 Kbp long transposase gene (*Trp1*) consisting of three exons (Figures 2B and S10B). More specifically, according to the Conserved Domain Database (CDD; http://www.ncbi.nlm.nih.gov/Structure/cdd/wrpsb.cgi), the predicted 566 aa long protein coded by *Trp1* (Figure S8) contains a transposase domain belonging to the DDE_TNP_1_7 superfamily (pfam 13843). It showed similarity (about 40%) with a number of uncharacterized hypothetical proteins of ascomycetes (i.e. XP_002144531; XP_002483130; XP_002150862) and with PiggyBac-like transposases (i.e. BAI68044; XP_002155850; NP_689808) of protist and metazoan species [44]. TblastN searches against the *T. melanosporum* TE database (http://mycor.nancy.inra.fr/IMGC/TuberGenome/blast.php) produced 13 hits with >35% aa identity. A BlastP search against the *T. melanosporum* genome database also showed similarity (41% aa identity) with the GSTUMT00002104001 protein annotated as PiggyBac-like TE. Searches of the Repbase (http://www.girinst.org/repbase/) using Censor (http://www.girinst.org/censor/index.php) also confirmed the similarity of this protein with transposases of PiggyBac-like TE (i.e. piggyBac-1_Mcir_1p from *Mucor circinelloides*).

Analysis of the sequences flanking the putative *Trp1* gene showed the presence of 52 bp long terminal inverted repeats (TIRs) (Figure S10A). However, the TTAA target site duplications (TSDs) typical of PiggyBac TE [45] were not found in Ti-tr1. Instead, the Ti-tr1 TIRs were integrated at TSDs represented by TA nucleotides (Figure S10B) which typifies different Class II TE families [46].

A second TE-like element was identified on the 3′ flanking region of the *T. indicum* Ti_CF10 *MAT1-2* idiomorph. It was present within a 1.868 bp long insertion specific to the idiomorph of this sample only and was named Ti_tr2 (Figure 2B, brown box). The Ti_tr2 element contained a 1.462 bp long putative gene (*Trp2*) coding for an hypothetical protein of 370 aa (Figure S9). BlastP analysis against the GenBank database showed similarity to transposase genes of ascomycetes such as the Tc1/mariner transposases of *Penicillium marneffei* (XP_002146479) and *Talaromyces stipitatus* (XP_002486812.1). Blast analysis against the *T. melanosporum* database showed low similarity (about 30% aa identity) with gene models (GSTUMT00012613001, GSTUMT00006493001, GSTUMT00007775001, GSTUMT00007054001, GSTUMT00012634001) annotated as transposases. Searches of the Repbase also confirmed the similarity of Ti_tr2 to Tc1/mariner TEs. The Ti_tr2 insertion sites were bordered by two 45 bp long TIRs integrated at TA TSDs (Figure S11).

The amino acid alignment of both *Trp1* and *Trp2* encoded hypothetical proteins with transposases from various organisms showed that they contained the highly conserved DDD/E catalytic triad. In addition, these two transposases showed a set of conserved aa positions (Figures S8 and S9), which typifies the PiggyBac and Tc1/mariner families, respectively [47].

## Discussion

In this study we show that *T. indicum,* the Asiatic truffle species which is the closest relative to the Périgord black truffle *T. melanosporum,* is heterothallic since its *MAT* locus is organized in two idiomorphs harbored by different strains. Because the sequences and the structure of the *MAT* idiomorphs differ between *T. indicum* strains belonging to different ITS classes, the presence of a complex of cryptic species is hypothesized. Comparison of *MAT* sequences between the *T. indicum* species complex and *T. melanosporum* also provides us with additional clues for inferring the evolutionary relationship between these black truffle species.

### *T. indicum* is Heterothallic

The scarce conservation of the *MAT* genes across different classes of fungi has been the main obstacle to identifying sexual genes in truffles by homologous cloning, and thus to deciphering their reproductive modes. Only recently, thanks to the sequencing of the *T. melanosporum* genome, the first *MAT* gene, namely *TmelMAT1-2-1*, has been mined from a truffle species [14]. In turn, the identification of this gene and its flanking genomic regions has allowed the second one, *TmelMAT1-1-1,* to be cloned in a different strain. This, coupled with the fact that none of the strains analyzed displayed both *MAT* genes, has provided clear cut evidence in support of the thesis that this black truffle is a heterothallic fungus [15]. Thus, *TmelMAT1-1-1* and *TmelMAT1-2-1* represent the bedrock for the identification of their orthologs from other fungi within the same genus by homologous cloning. To this end, we employed PCR primers designed on the most conserved domains, namely the α and HMG domains, of the two *T. melanosporum MAT* genes, to amplify and clone the *MAT* genes from *T. indicum.* All *T. indicum* sequences obtained using *MAT* domain specific primer pairs showed high similarity to both *T. melanosporum MAT,* regardless of the morphotypes of the samples analysed. Consistent with a genomic organization that typifies heterothallic fungi, none of the Chinese black truffles screened displayed both *MAT* genes when PCR was carried out to amplify preferentially the DNA contributed by the gleba, the structure of truffle fruit bodies known to be of uniparental origin [12], [13]. Equally consistent with heterothallism is the evidence that amplicons specific to both *MAT* genes have been conversely obtained when PCR was carried out to allow the amplification of the DNA contributed by the spores (see below). Strikingly, these analyses also prove that the gleba of the Chinese truffles can be formed by strains carrying either the *MAT1-2-1* or the *MAT1-1-1* gene, as previously observed in *T. melanosporum* [13].

### The *MAT* Genes and the Structure of *MAT* Idiomorphs Differed among *T. indicum* Samples

*T. indicum* is considered a highly polymorphic species, as fruit bodies differing in the morphological traits of their ascospores have been described [21], [22], [23], [24]. Morphological analyses of samples of different geographical origin have, indeed, shown a high level of overlapping characters, making it very difficult to distinguish putative different species within this complex ([26], this study). A large-scale screening using ITS/RFLP analyses, of *T. indicum* samples exhibiting different morphotypes has revealed a high level of genetic diversity with the presence of three different genotypes (haplotypes) named *T. indicum* A, B1 and B2 within these samples [27]. Subsequently, phylogenetic studies based on the sequences of the LSU and ITS of the rDNA region, and of other phylogenetically informative loci such as β-tubulin, 1α-elongation factor and Protein kinase C (PKC), have converged to show the presence within *T. indicum* of at least two distinctive clusters referred to as *T. indicum*_A and *T. indicum*_B [26], [28], [29], [30], [31], [32], [34], [48]. However, whether these two *T. indicum* classes correspond to distinctive cryptic species or merely represent highly-structured populations belonging to the same species has yet to be elucidated [26], [31].

The *MAT* locus is typically a non recombining region and many studies have shown that *MAT* genes, in particular in heterothallic species, are under relaxed purifying selection or even under positive selection and have high evolutionary rates [49], [50], [51], [52]. Fast-evolving genes in the *MAT* locus are thus good candidates for tackling taxonomic and phylogenetic questions and for better delineating species boundaries within the *Tuber* genus. To this end, we have isolated and sequenced the entire *T. indicum* mating type idiomorphs from ascocarps belonging to the three ITS classes (A, B1 and B2) reported by Paolocci et al. [27]. More specifically, in order to compare their sequences across genetically different samples we have isolated the two idiomorphs from the same ascocarp to ensure the cloning of each pair of idiomorphs from the same biological species. This has been achieved by increasing the number of PCR cycles to allow the DNA isolated from the spores of each ascocarp to be amplified above the detection limit, although to a much lesser extent than the DNA isolated from the surrounding gleba.

The sequences of both *MAT* genes are very similar in the *T. indicum*_B samples (Ti_U983 and Ti_U986): only two synonymous substitutions are in fact present in *MAT1-1-1,* and a single non-synonymous substitution is present in *MAT1-2-1.* Conversely, *T. indicum*_A displays many non-synonymous substitutions in both *MAT1-1-1* (8) and *MAT1-2-1* (13–14) with respect to their orthologs from the *T. indicum*_B samples, with only a few (1–2) of them occurring within the α-box and HMG-box regions (Figure 3). These polymorphisms between the *T. indicum*_A and *T. indicum*_B samples are also evident in the genomic regions flanking each of the *MAT* genes (Figure 2). Notably, the *T. indicum*_A *MAT1-2* idiomorph differed from those of the *T. indicum*_B samples for a deletion within the gene named *Ti_orf3*. This deletion does not disrupt the ORF of the gene but, by changing the number of ANK binding domains, may affect gene function [53]. Length difference of the *MAT1-2* idiomorph is also observed in the *T. indicum*_B samples. However this is due to the insertion of a TE element in Ti_U983 (see below).

With regard to the *MAT1-1* idiomorphs, the main polymorphism resides in a region of about 750 bp (Figure 2, region “e” of Ti_CF10) with a sequence totally different between *T. indicum*_A and *T. indicum*_B samples.

*MAT* genes are functional markers intimately linked to speciation events. Comparative analyses of the sequence and organization of the *MAT* genes and idiomorphs suggest significant divergences between *T. indicum* truffles displaying the ITS class A and those displaying classes B1 and B2. This is therefore interpreted as an additional clue in support of the thesis that there are at least two cryptic species within the *T. indicum* complex. On these grounds, the present study provides a methodological approach (i.e. the amplification of both *MAT* genes/idiomorphs from the same ascocarp) and sequence information to carry out a large-scale screening of *T. indicum* truffles of different ITS classes by *MAT*-based markers. This screening will be instrumental to further verifying the hypothesis that the *T. indicum*_A and *T. indicum*_B samples belong to two different species. Under this assumption, in fact, no *T. indicum*_A sample is expected to show traits that feature the two *MAT* idiomorphs of the B classes. In contrast, some *T. indicum*_B1 samples are expected to display one or both *MAT* idiomorphs displayed by *T. indicum*_B2 samples and vice versa.

### TE Elements in *T. indicum MAT* Locus

Extensive length difference has been observed among the *MAT1-2* regions of *T. indicum* samples. More specifically, the *MAT1-2* idiomorph of *T. indicum*_B1 sample (Ti_U983) is longer than that of *T. indicum*_B2 (Ti_U986) due to the presence of a Class II TE, named Ti_tr1. A class II TE, named Ti_tr2, is also present in the *MAT1-2* region of *T. indicum*_A. However, it is located outside of the idiomorph, in its 3′ flanking region.

Class II TEs comprise several superfamilies which are defined on the basis of terminal inverted repeat (TIR) sequences, size and/or sequence of the TSD and structure and conservation of catalytic domains of the transposase protein [47], [54]. Both Ti_tr1 and Ti_tr2 have a “TA” TSD which typifies the Tc1/mariner superfamily. Blast similarity searches using the *Trp2* encoded putative transposase protein of Ti_tr2 as a query also return TEs of the Tc1/mariner superfamily as best hits. Conversely, the classification of Ti_tr1 remains unclear because its transposase appears to be related to TEs of the PiggyBac superfamily, which is characterized by a different TSD (i.e. TTAA) [55]. The scarce similarity between TIRs sequences of Ti_tr1 and Ti_tr2 (Figure S12) and the phylogenetic tree relative to *Trp1* and *Trp2* encoded putative transposases (Figure S13) confirm that these two TEs are unrelated to each other.

Tc1/mariner TEs are widespread in fungi [46], conversely PiggyBac TEs are mainly found in animals and insects, more rarely in fungi [45]. However, TEs similar to Ti_tr1 have been identified and annotated as PiggyBac in the *T. melanosporum* genome [14].

The presence of Class I and II TEs within or linked to the mating type region is a common feature of many filamentous ascomycetes [51], [52], [56], [57], [58], [59], [60]. Because of its low recombination rate, the accumulation of TEs and other mutations in the *MAT* locus is an expected result [61]. In turn, the accelerated divergence of mating types may promote speciation by determining reproductive isolation between individuals [59]. Thus, TEs within or linked to the *MAT* locus of *T. indicum* may have had a crucial role in the diversification of this species complex. In addition, TE insertion, by generating sites for unequal crossing over, may trigger gross rearrangements in fungal genomes, which if targeted on the *MAT* locus may induce changes in their lifestyle [59]. Indeed, Gioti et al. [62] have attributed the transition from heterothallism to homothallism during the evolutionary history of *Neurospora* to retrotransposons.

Based on these considerations and the finding that TE elements have been detected within or linked to the *MAT* locus of black truffle species, the occurrence of events over the evolutionary scale of mating system transition in these fungi cannot be excluded. It will be interesting therefore to assess whether heterothallism and the presence of *MAT*-linked TEs are common features of all *Tuber* spp.

### Comparison of *T. indicum* and *T. melanosporum MAT* Loci Provides Insights into the Evolution of Black Truffles

Comparative analyses reveal a similar structure of both *MAT* idiomorphs between *T. indicum* and *T. melanosporum*. The *MAT1-1* idiomorph of both species contains a single gene, *MAT1-1-1*, in the same position and orientation. The *T. indicum* and *T. melanosporum MAT1-1-1* genes share the same intron/exon structure with the predicted proteins differing for 9-10 amino acids. In a previous study, Rubini et al. [15] showed that the second intron of *MAT1-1-1* is differentially spliced in the *T. melanosporum* fruit body to produce two transcripts coding for proteins of 319 and 399 amino acids. Here we show the presence of a single transcript coding for a putative protein of 319 aa in both the *T. indicum*_A and *T. indicum*_B fruit bodies. So, whether alternative splicing of *MAT1-1-1* is a stage or species-specific phenomenon has yet to be elucidated.

Compared to *MAT1-1,* the structure of *MAT1-2* idiomorphs of *T. indicum* and *T. melanosporum* is more divergent: in addition to *MAT1-2-1* gene which shares the same structure and similar sequence, a putative gene (*Ti_orf3*) coding for an ANK domain containing protein is present in the *MAT1-2-1* of *T. indicum* samples only. *T. melanosporum* displays a large deletion in this region that encompasses a large portion of the *Ti_orf3* gene.

However, a highly degenerated relict sequence corresponding to the 3′ end of *Ti_orf3* can still be identified in the MAT1-2 of *T. melanosporum*.

It is worth mentioning that an ANK-containing gene has been also identified in the *MAT* idiomorph of *Rhizopus oryzae*, a species belonging to a basal group of fungi, the Mucorales [63]. However, this *R. oryzae* gene contains two additional domains (BTB and RCC1) that are not present in *T. indicum Ti_orf3*, suggesting that these two ANK-containing genes are not orthologs.

Although additional experiments are required to evaluate whether the *Ti_orf3* gene is functional in *T. indicum* and whether it is involved in any phase of sexual reproduction, it may be helpful for taxonomic and phylogenetic studies. The presence of the *Ti_orf3* relict sequence in *T. melanosporum* suggests, in fact, that it is an ancestral gene that has been likely lost in *T. melanosporum* after the divergence from the common ancestor of these two black truffle species. This is in agreement with the hypothesis of vicariance for explaining the allopatric isolation of *T. indicum* from *T. melanosporum* populations in Asia and Europe [34].

Bonito et al. [64], based on dispersal-vicariance analysis, have recently suggested a North American origin for *T. melanosporum*, hypothesizing that European and Asiatic black *Tuber* species originate from long-range dispersal events (i.e. migration via the Beringia and/or Thulean North Atlantic Land Bridges). Sequencing of *MAT* regions of more Asian, European and North American species (e.g. *T. brumale, T. regimontanum, T. pseudoexcavatum, T. formosanum*) belonging to the *T. indicum* - *T. melanosporum* clade [34], [48], [64] is required to deepen our knowledge of the evolutionary history of this fungal lineage.

Comparative analyses of the structure and organization of the sex locus in basal lineages of filamentous Ascomycetes (Pezizomycotina), such as members of the Pezizomycetes, may help to investigate the mechanisms underlying fungal mating type evolution. On these grounds, the comparison of the two *T. indicum* idiomorphs among them and with those of *T. melanosporum* has revealed the presence of homologous sequences arranged in an opposite orientation. This is especially true in *T. indicum* where these shared sequences represent approximately 50% of the entire idiomorphic region (Figure 2). The presence in opposite orientation of sequences shared between the idiomorphs fits nicely with the hypothesis that inversions at chromosomal loci containing genes for sex determination may have represented a mechanism that has driven the divergence (and rearrangement) of the sex locus in ascomycetes and other organisms [59]. The presence in the *MAT* locus of *Tuber* species of TE elements (see above) reinforces such a thesis.

### Are *T. indicum* and *T. melanosporum* Potentially Sexually Compatible?

Asiatic black truffles have been imported into Europe since the early 1990s and frequently sold in local markets mixed with the more highly-prized Périgord black truffle [17], [40]. Besides the fraudulent commercialization of Asiatic black truffles, the deliberate or accidental use of *T. indicum* ascocarps to inoculate host plants for establishing truffle orchards in Europe may represent a serious ecological threat for the indigenous populations of *T. melanosporum*. Incidentally, the introduction of *T. indicum* in *T. melanosporum* truffle orchards in Italy as well as in non-European countries (e.g. the USA) has been reported [48], [65].

It is worth noting that for both *MAT* genes the divergence level between the two *T. indicum* classes (A and B) is similar to that between *T. indicum* and *T. melanosporum* (Table S3). Thus, if hybridization between genetically different *T. indicum* individuals from sympatric populations were discovered, sexual compatibility with *T. melanosporum* could not be ruled out. It is interesting to note that interspecific mating and hybridization has been reported for fungi such as *Ophiostoma* spp., *Heterobasidion* spp., *Melampsora* spp. and *Microbotryum* spp. [66], [67], [68], [69], [70]. In some of these fungi (e.g. *Microbotryum*) hybridization has occurred through secondary contact following initial divergence in allopatry [70].

In Ascomycetes, mating compatibility and mutual recognition between strains of opposite mating type are mediated by the pheromone-receptor system [52]. In a wide range of organisms it has been demonstrated that proteins under sex-biased expression such as gamete recognition proteins evolve rapidly [71], [72]. A fundamental consequence of the rapid evolution of interacting reproductive proteins is the potential to generate reproductive isolation between populations or species [72], [73]. Although rapid evolution appears to be the case for fungal *MAT* genes, this trend has not been ascertained for ascomycete pheromones [49], [74], [75]. The distinct pheromones of certain yeast species appear to offer some level of species specificity, it is therefore conceivable that reproductive barriers between some ascomycetes may stem from differences in their pheromone peptide and pheromone receptor sequences.

The α-pheromone and both pheromone receptors have been identified in *T. melanosporum* genome [14], [15]. Work is in progress to isolate their orthologs from *T. indicum* and perform functional analyses using yeast complementation assays [76]. This will allow us to assess for the level of conservation of these genes across *Tuber* spp. and test whether the pheromones of one truffle species are recognized by the receptors of the other(s) within *T. indicum* species complex and between species of this complex and *T. melanosporum*.

## Conclusions

*T. indicum* is the first truffle species which is non-endemic in Europe for which the *MAT* locus and thus the reproductive mode have been characterized. This locus has helped us to shed more light on the taxonomy and phylogenetic relatedness within and between black truffles of different geographical provenance. In light of their phylogenetic relatedness and heterothallism, we cannot exclude breeding between *T. melanosporum* and Chinese truffles. In turn, if geographical barriers have thus far been the only impediment for their crossing, the introduction of one species into the geographical range of the other could have potentially extremely detrimental effects in terms of erosion of their biodiversity and specificity. This is particularly true for the less competitive and highly prized *T. melanosporum*. In addition, recent studies have documented a skewed representation of both mating types on host plants colonized by *T. melanosporum* to suggest a possible mating type-dependent distributional pattern of its strains [77], [78]. Thus it becomes intriguing and of both fundamental and applied relevance to assess the distributional pattern of strains with opposite mating types also in *T. indicum* producing sites.

## Supporting Information

Figure S1 **Morphology of the ascospores of** ***T. indicum_*** **A ascocarps.** a: Ti_CF4; b: Ti_CF10; c: Ti_CF11; d: Ti_CF3; e: Ti_D3; f: Ti_C24; g: Ti_C4; h: Ti_C21; i: Ti_C66; l: Ti_CF5; m: Ti_D9; n: Ti_C37; o: Ti_C55; p: Ti_C69; q: Ti_C47; r: Ti_C57; s: Ti_CU3.
(DOC)

Figure S2 **Morphology of the ascospores of** ***T. indicum_*** **B1 ascocarps.** a: Ti_CF14; b: Ti_D6; c: Ti_D15; d: Ti_D23; e: Ti_D31; f: Ti_D54; g: Ti_F4; h: Ti_RIBC; i: Ti_C1; l: Ti_C20; m: Ti_F2; n: Ti_U982; o: Ti_C22; p: Ti_C30; q: Ti_C40; r: Ti_U983; s: Ti_F3.
(DOC)

Figure S3 **Morphology of the ascospores of** ***T. indicum_*** **B2 ascocarps.** a: Ti_CF2; b: Ti_CF7; c: Ti_LI8; d: Ti_C2; e: Ti_C8; f: Ti_C18; g: Ti_C29; h: Ti_C31; i: Ti_C38; l: Ti_C61; m: Ti_U986; n: Ti_C3; o: Ti_C9; p: Ti_C15; q: Ti_C27
(DOC)

Figure S4 **Schematic representation of the PCR-based strategy used to isolate the** ***T. indicum MAT1-1*** **(A) and** ***MAT1-2*** **(B) idiomorphs.** The white arrowed boxes indicate the *MAT1-1-1* and *MAT1-2-1* gene, respectively. Black arrows indicate the annealing sites of primers numbered as in Table S2. The black lines at the bottom of the figures indicate the PCR amplicons obtained with the different primer combinations; the name of *T. indicum* sample and the approximate length of the PCR amplicon are given in brackets.
(DOC)

Figure S5 **PCR amplification of** ***T. indicum MAT*** **idiomorphs with the primer pair i7/i10.** Lane 1 Gene Ruler DNA Ladder Mix (Fermentas International Inc., Waltham, MA, USA); Lane 2 Ti_U983; Lane 3 Ti_CF10; Lane 4 Ti_U986; Lane 5 negative control, no DNA template.
(DOC)

Figure S6 **Nucleotide alignment of** ***T. indicum*** **and** ***T.*** ***melanosporum MAT1-1-1*** **genes.** Introns are shown in bold type.
(DOC)

Figure S7 **Nucleotide alignment of** ***T. indicum*** **and** ***T.*** ***melanosporum MAT1-2-1*** **genes.** Introns are shown in bold type.
(DOC)

Figure S8 **Alignment of** ***Trp1*** **encoded hypothetical protein with Piggybac transposases.** Black arrows indicate the conserved DDD catalytic triad; red arrows indicate conserved amino acid residues as reported by Youan and Wessler [47]. The default ClustalX color code is used.
(DOC)

Figure S9 **Alignment of** ***Trp2*** **encoded hypothetical protein with TC1/mariner transposases.** Black arrows indicate the conserved DD(D/E) catalytic triad; red arrows indicate conserved amino acid residues as reported by Youan and Wessler [47]. The default ClustalX color code is used.
(DOC)

Figure S10 **Nucleotide sequence and structure of Ti_tr1 transposon.** A) Nucleotide alignment of Ti_tr1 TIR. B) Organization of the Ti_tr1 transposon. TIRs are indicated in Bold; the TSDs in red; the putative transposase CDS in blue; the putative start and stop codons are underlined.
(DOC)

Figure S11 **Nucleotide sequence and structure of Ti_tr2 transposon.** A) Nucleotide alignment of Ti_tr2 TIR. B) Organization of the Ti_tr2 transposon. TIRs are indicated in Bold; the TSDs in red; the putative transposase CDS in blue; the putative start and stop codons are underlined.
(DOC)

Figure S12 **Nucleotide alignment of Ti_tr1 and Ti_tr2 TIRs.**
(DOC)

Figure S13 **Phylogenetic tree showing the relationship of** ***T. indicum Trp1*** **and** ***Trp2*** **encoded transposases with those of others related TEs.** Phylogenetic tree was inferred with the Neighbor-Joining method and the Poisson distance model using the software Mega v. 5.05. Numbers near the branches indicate the bootstrap values (percentage of 1000 replicates).
(DOC)

Table S1 ***T. indicum*** **samples analyzed.** A, B1, B2 indicates the three ITS/RFLP patterns obtained with the *Rsa*1 endonuclease.
(DOC)

Table S2 **List of primers used in this study.**
(DOC)

Table S3 **Nucleotide differences between** ***T. indicum*** **and** ***T. melanosporum MAT1-2-1*** **and** ***MAT1-1-1*** **genes.**
(DOC)
