# Supplementary material for: Mating Type Locus of Chinese Black Truffles Reveals Heterothallism and the Presence of Cryptic Species within the T. indicum Species Complex
Source: PLoS One. 2013 Dec 16;8(12):e82353. doi: 10.1371/journal.pone.0082353 (PMC3864998; doi:10.1371/journal.pone.0082353)

**Figure S1 Morphology of the ascospores of *T. indicum_*A ascocarps.** a: Ti_CF4; b: Ti_CF10; c: Ti_CF11; d: Ti_CF3; e: Ti_D3; f: Ti_C24; g: Ti_C4; h: Ti_C21; i: Ti_C66; l: Ti_CF5; m: Ti_D9; n: Ti_C37; o: Ti_C55; p: Ti_C69; q: Ti_C47; r: Ti_C57; s: Ti_CU3.


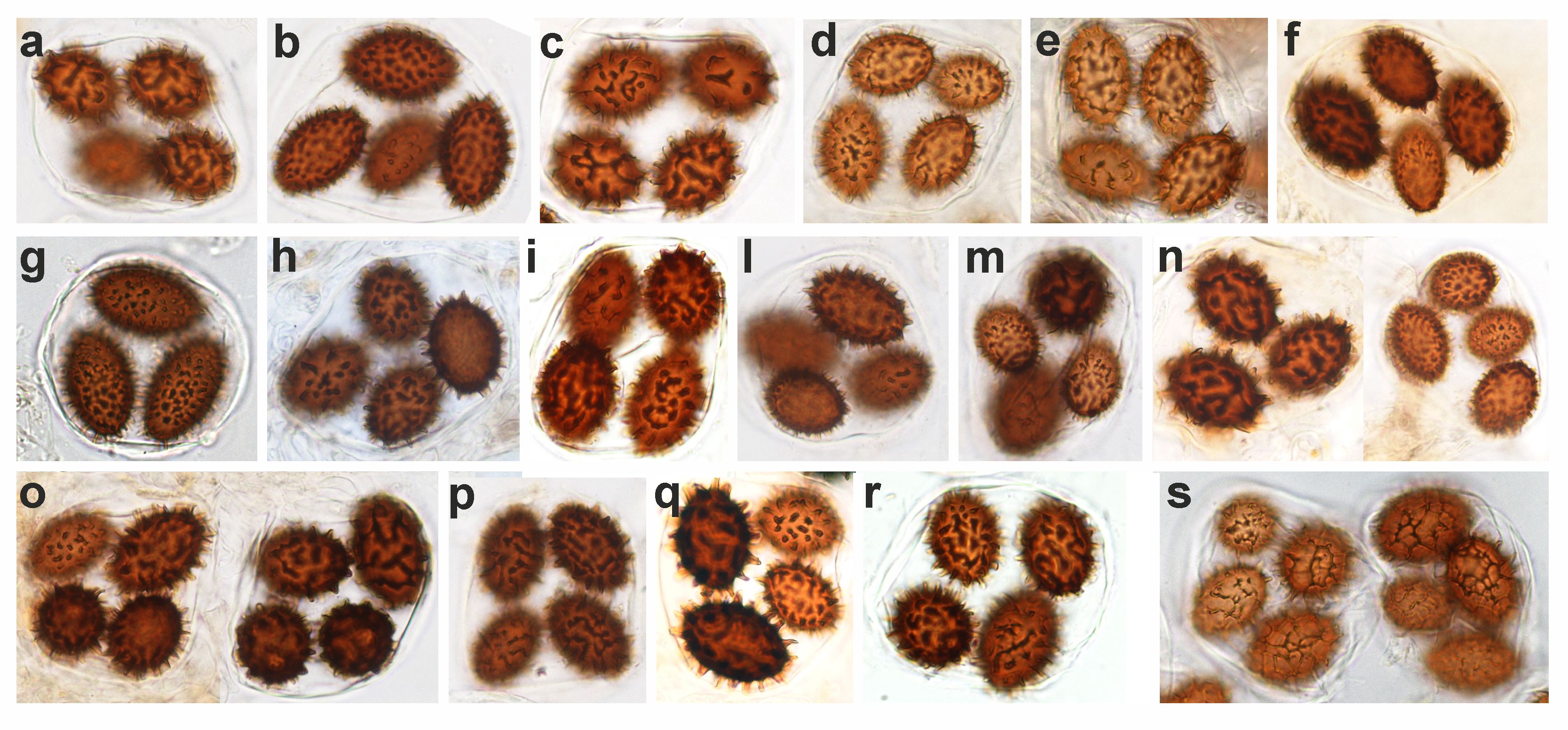

Supplement: Figure S1 — Morphology of the ascospores of T. indicum_ A ascocarps. a: Ti_CF4; b: Ti_CF10; c: Ti_CF11; d: Ti_CF3; e: Ti_D3; f: Ti_C24; g: Ti_C4; h: Ti_C21; i: Ti_C66; l: Ti_CF5; m: Ti_D9; n: Ti_C37; o: Ti_C55; p: Ti_C69; q: Ti_C47; r: Ti_C57; s: Ti_CU3. (DOC) [file pone.0082353.s001.doc]
