# Supplementary material for: Mating Type Locus of Chinese Black Truffles Reveals Heterothallism and the Presence of Cryptic Species within the T. indicum Species Complex
Source: PLoS One. 2013 Dec 16;8(12):e82353. doi: 10.1371/journal.pone.0082353 (PMC3864998; doi:10.1371/journal.pone.0082353)

**Figure S2 Morphology of the ascospores of *T. indicum_*B1 ascocarps.** a: Ti_CF14; b: Ti_D6; c: Ti_D15; d: Ti_D23; e: Ti_D31; f: Ti_D54; g: Ti_F4; h: Ti_RIBC; i: Ti_C1; l: Ti_C20; m: Ti_F2; n: Ti_U982; o: Ti_C22; p: Ti_C30; q: Ti_C40; r: Ti_U983; s: Ti_F3.


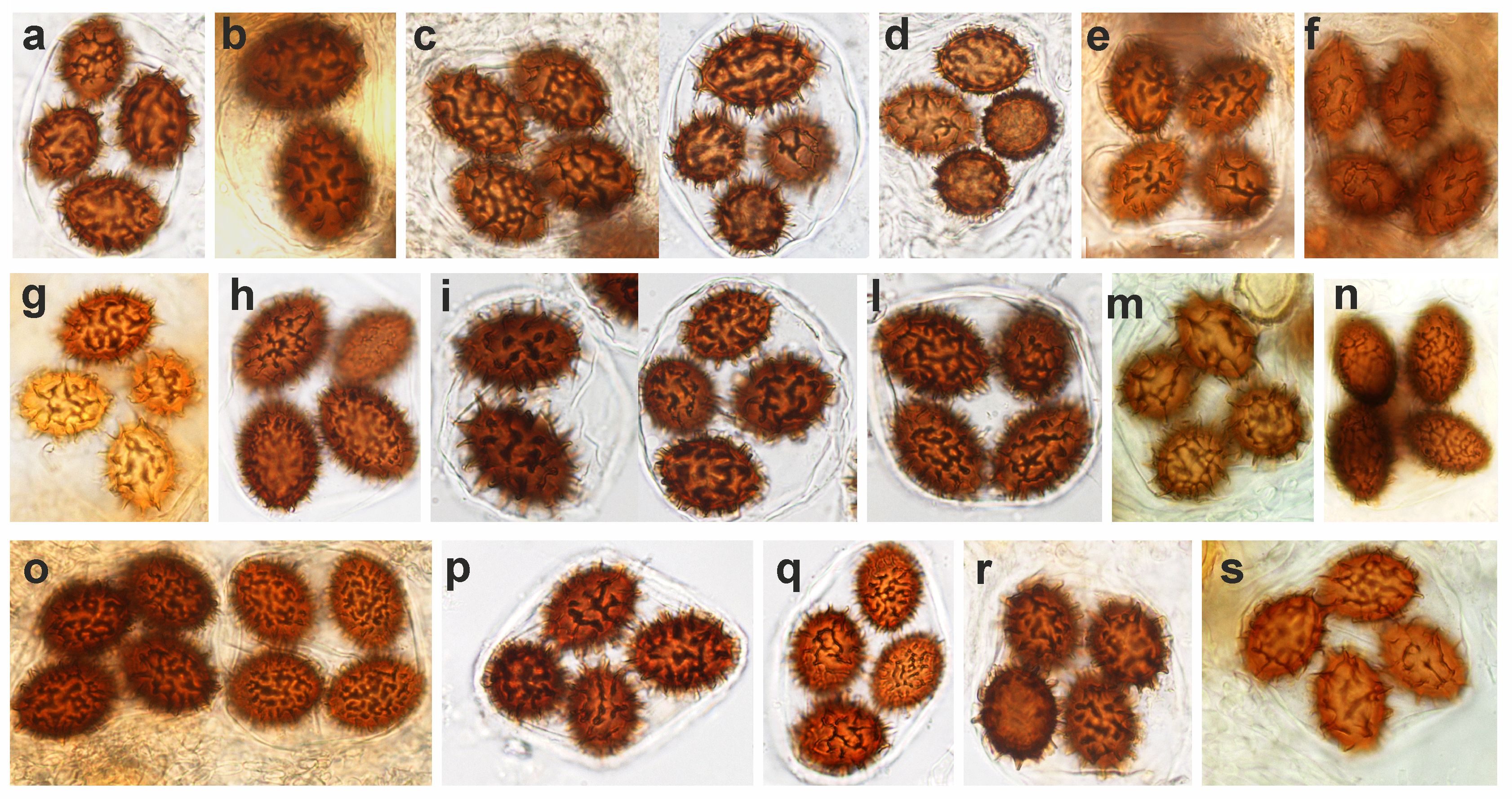

Supplement: Figure S2 — Morphology of the ascospores of T. indicum_ B1 ascocarps. a: Ti_CF14; b: Ti_D6; c: Ti_D15; d: Ti_D23; e: Ti_D31; f: Ti_D54; g: Ti_F4; h: Ti_RIBC; i: Ti_C1; l: Ti_C20; m: Ti_F2; n: Ti_U982; o: Ti_C22; p: Ti_C30; q: Ti_C40; r: Ti_U983; s: Ti_F3. (DOC) [file pone.0082353.s002.doc]
