# Supplementary material for: Mating Type Locus of Chinese Black Truffles Reveals Heterothallism and the Presence of Cryptic Species within the T. indicum Species Complex
Source: PLoS One. 2013 Dec 16;8(12):e82353. doi: 10.1371/journal.pone.0082353 (PMC3864998; doi:10.1371/journal.pone.0082353)

**Figure S3 Morphology of the ascospores of *T. indicum_*B2 ascocarps.** a: Ti_CF2; b: Ti_CF7; c: Ti_LI8; d: Ti_C2; e: Ti_C8; f: Ti_C18; g: Ti_C29; h: Ti_C31; i: Ti_C38; l: Ti_C61; m: Ti_U986; n: Ti_C3; o: Ti_C9; p: Ti_C15; q: Ti_C27


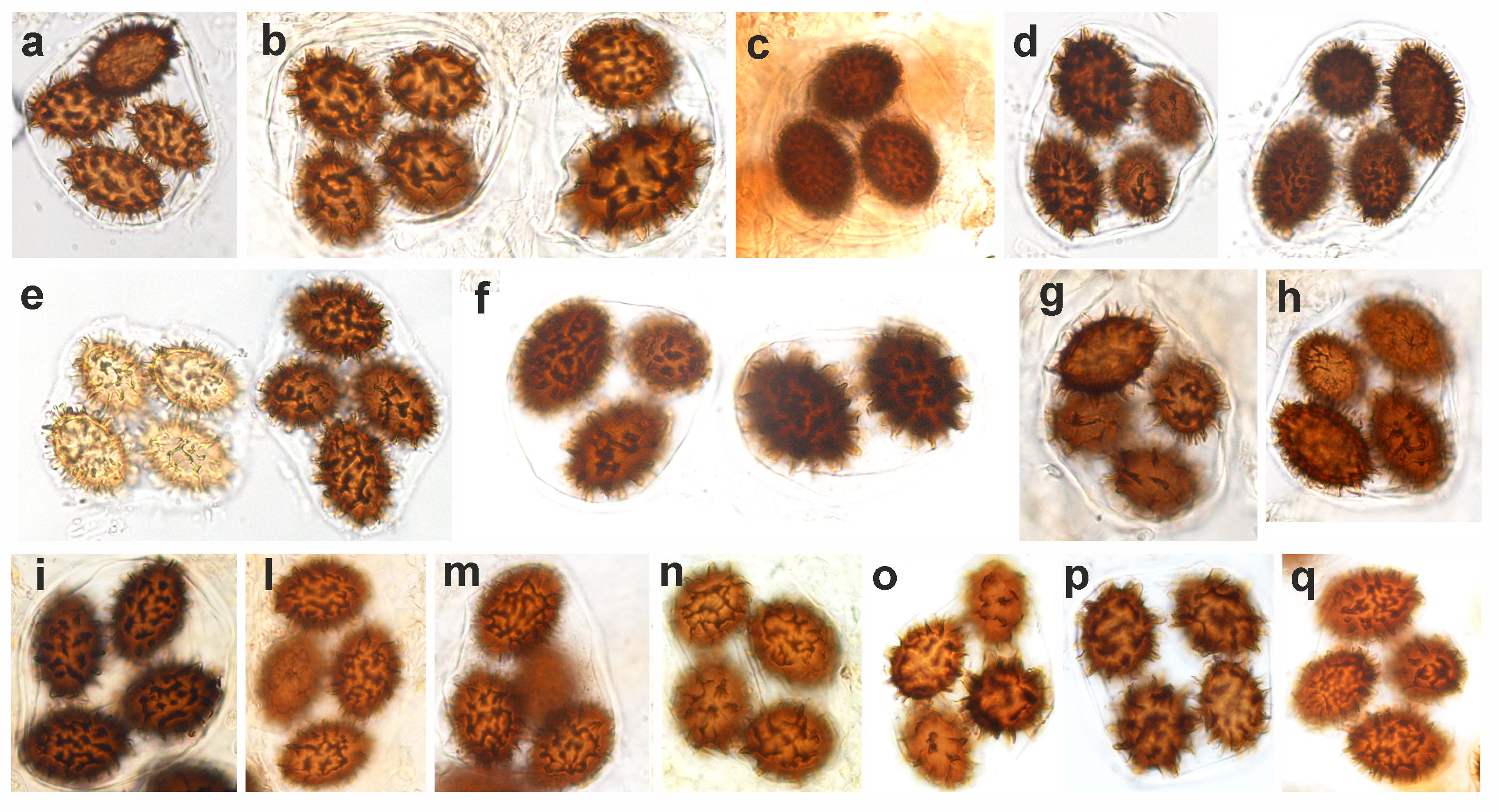

Supplement: Figure S3 — Morphology of the ascospores of T. indicum_ B2 ascocarps. a: Ti_CF2; b: Ti_CF7; c: Ti_LI8; d: Ti_C2; e: Ti_C8; f: Ti_C18; g: Ti_C29; h: Ti_C31; i: Ti_C38; l: Ti_C61; m: Ti_U986; n: Ti_C3; o: Ti_C9; p: Ti_C15; q: Ti_C27 (DOC) [file pone.0082353.s003.doc]
