# Supplementary material for: Mating Type Locus of Chinese Black Truffles Reveals Heterothallism and the Presence of Cryptic Species within the T. indicum Species Complex
Source: PLoS One. 2013 Dec 16;8(12):e82353. doi: 10.1371/journal.pone.0082353 (PMC3864998; doi:10.1371/journal.pone.0082353)

**Figure S4 Schematic representation of the PCR-based strategy used to isolate the *T. indicum* MAT1-1 (A) and MAT1-2 (B) idiomorphs.** The white arrowed boxes indicate the *MAT1-1-1* and *MAT1-2-1* gene, respectively. Black arrows indicate the annealing sites of primers numbered as in Table S2. The black lines at the bottom of the figures indicate the PCR amplicons obtained with the different primers combinations; the name of *T. indicum* sample and the approximate length of the PCR amplicon are given in brackets.


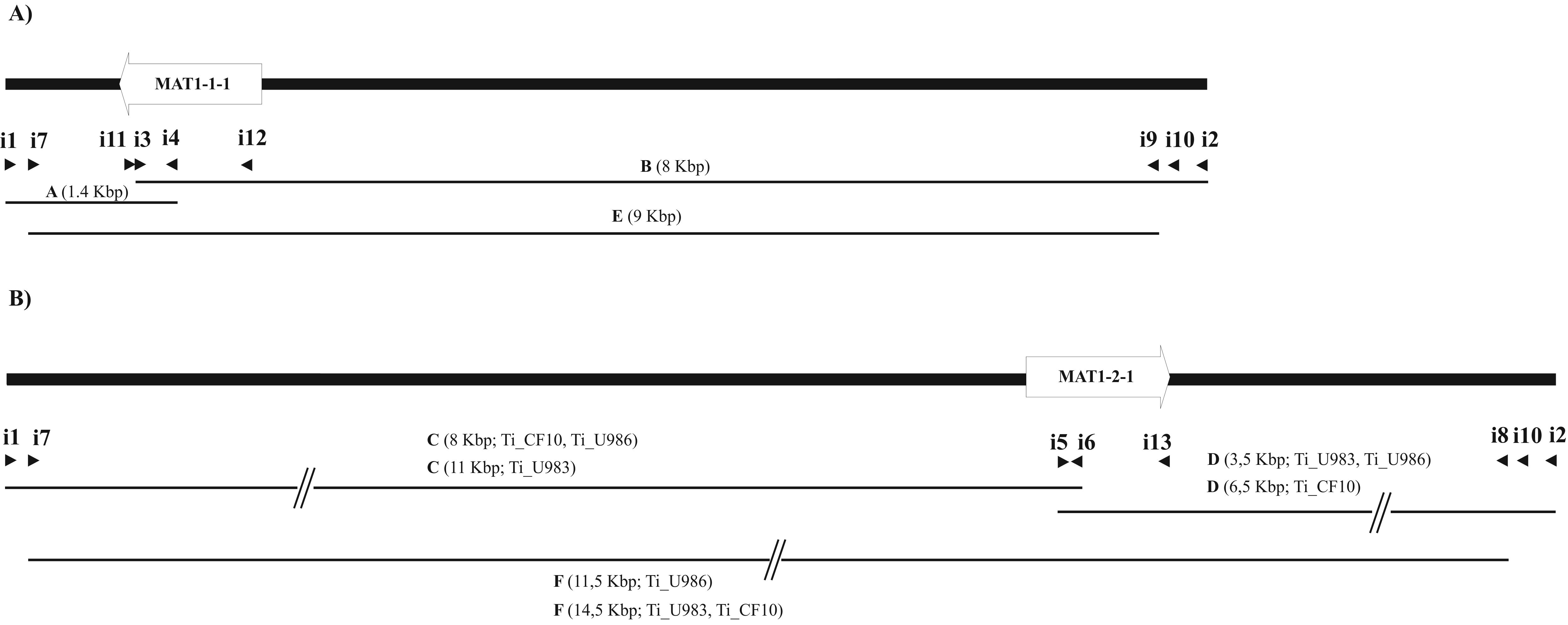

Supplement: Figure S4 — Schematic representation of the PCR-based strategy used to isolate the T. indicum MAT1-1 (A) and MAT1-2 (B) idiomorphs. The white arrowed boxes indicate the MAT1-1-1 and MAT1-2-1 gene, respectively. Black arrows indicate the annealing sites of primers numbered as in Table S2. The black lines at the bottom of the figures indicate the PCR amplicons obtained with the different primer combinations; the name of T. indicum sample and the approximate length of the PCR amplicon are given in brackets. (DOC) [file pone.0082353.s004.doc]
