# Supplementary material for: Mating Type Locus of Chinese Black Truffles Reveals Heterothallism and the Presence of Cryptic Species within the T. indicum Species Complex
Source: PLoS One. 2013 Dec 16;8(12):e82353. doi: 10.1371/journal.pone.0082353 (PMC3864998; doi:10.1371/journal.pone.0082353)
Supplement: Figure S5 — PCR amplification of T. indicum MAT idiomorphs with the primer pair i7/i10. Lane 1 Gene Ruler DNA Ladder Mix (Fermentas International Inc., Waltham, MA, USA); Lane 2 Ti_U983; Lane 3 Ti_CF10; Lane 4 Ti_U986; Lane 5 negative control, no DNA template. (DOC) [file pone.0082353.s005.doc]

**Figure S5 PCR amplification of *T. indicum* *MAT* idiomorphs with the primer pair i7/i10**. Lane 1 Gene Ruler DNA Ladder Mix (Fermentas International Inc., Waltham, MA, USA); Lane 2 Ti_U983; Lane 3 Ti_CF10; Lane 4 Ti_U986; Lane 5 negative control, no DNA template.

**1 2 3 4 5**

**
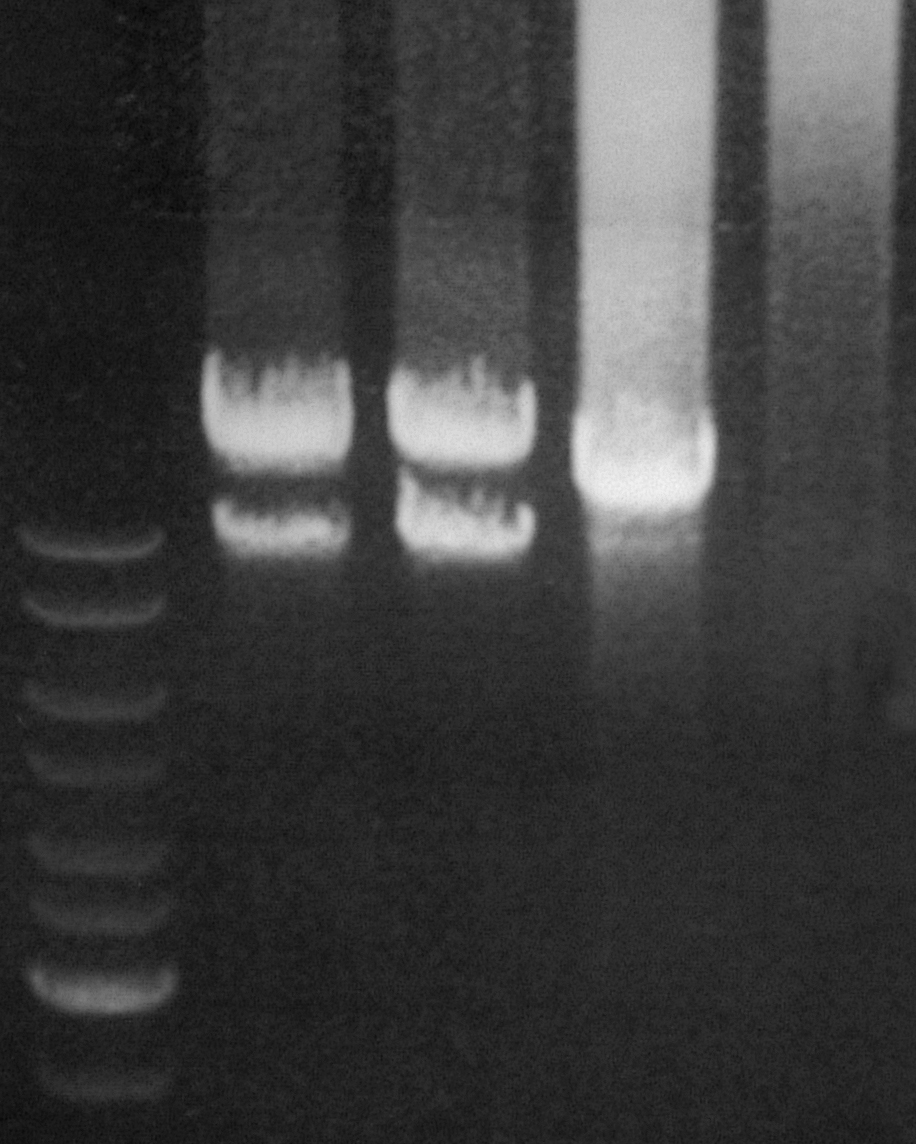
**

**10 Kbp**

**3 Kbp**
