# Supplementary material for: Mating Type Locus of Chinese Black Truffles Reveals Heterothallism and the Presence of Cryptic Species within the T. indicum Species Complex
Source: PLoS One. 2013 Dec 16;8(12):e82353. doi: 10.1371/journal.pone.0082353 (PMC3864998; doi:10.1371/journal.pone.0082353)
Supplement: Figure S6 — Nucleotide alignment of T. indicum and T. melanosporum MAT1-1-1 genes. Introns are shown in bold type. (DOC) [file pone.0082353.s006.doc]

**Figure S6 Nucleotide alignment of *T. indicum* and *T. melanosporum* *MAT1-1-1* genes**. Introns are shown in bold type underlined.

10 20 30 40 50 60 70 80 90 100 110 120 130 140 150 160 170

....|....|....|....|....|....|....|....|....|....|....|....|....|....|....|....|....|....|....|....|....|....|....|....|....|....|....|....|....|....|....|....|....|....|

**Tmel-MAT111**  ATGCGTAATGTTACCTTGGGATGCGCCTTGTACCTTTCGGACCGTGGGTACATTCTCATGCAAGACAGGATCGGGTGTATCTGGGTCCAGCACGAGAACTCCCCCATTATCGTTCAACCCCCCGGTGGTGTCACTTTCACTGTCAGCGGCGAACTCTTGCCCTACGACAA 170

**Ti_U986-MAT111** ATGCGTAATGCTACCTTGGGATGCGCCTTGTACCTTTCGGACCGTGGGTACATTCTCATGCAAGACAGGATCGGGTGCATCTGGGTCCAACACGAGAACTCCCCCATCATCGTTCAACCCCCCGGTGGTGTCACTTTCACTGTCAGCGGCGAACTCTTGCCCTACGACAA 170

**TI_U983-MAT111** ATGCGTAATGCTACCTTGGGATGCGCCTTGTACCTTTCGGACCGTGGGTACATTCTCATGCAAGACAGGATCGGGTGCATCTGGGTCCAACACGAGAACTCCCCCATTATCGTTCAACCCCCCGGTGGTGTCACTTTCACTGTCAGCGGCGAACTCTTGCCCTACGACAA 170

**Ti_CF10-MAT111** ATGCGCAATGCTACCTTGGGATGCGCCTTGTACCTTTCGGACCGTGGGTACATCCTCATGCAAGACAGGATCGGGTGCATCTGGGTCCAACATGAGAACTCCCCCATTATCGTTCAACCCCCCGGTGGTGTCACTTTCACTGTCAGCGGCGAACTCTTGCCCTATGACAA 170

180 190 200 210 220 230 240 250 260 270 280 290 300 310 320 330 340

....|....|....|....|....|....|....|....|....|....|....|....|....|....|....|....|....|....|....|....|....|....|....|....|....|....|....|....|....|....|....|....|....|....|

**Tmel-MAT111**  GTGGCTCGGTGCAATTCAATCAACCGTCCGTCATCAAGGGCGAGTCCCCCTCGACCCCGAAGTCCCTTATGTTGTGTCGGTTCTGCGTTTGTATTGCGGATACTATAAACCTGGACAGCCGTGGACGGCCCAGGATGCCACTATTCCGGCTGTTATCGTCAATGGTGATG 340

**Ti_U986-MAT111** GTGGCTTGGTGCAATTCAATCAACCGTCCGTCATCGAGGGCGAGTCCCCCTCGACCCCGAAGTCCCTTATGTTGTGTCGGTTCTACGTTTGTATTGCGGATACTATAAACCTGGACAACCGTGGACGGCCCAGGATGCCACTATTCCGGCTGTTATCGTCAATGGTGATG 340

**Ti_U983-MAT111** GTGGCTTGGTGCAATTCAATCAACCGTCCGTCATCGAGGGCGAGTCCCCCTCGACCCCGAAGTCCCTTATGTTGTGTCGGTTCTACGTTTGTATTGCGGATACTATAAACCTGGACAGCCGTGGACGGCCCAGGATGCCACTATTCCGGCTGTTATCGTCAATGGTGATG 340

**Ti_CF10-MAT111** GTGGCTCGGTGCAATTCAATCAACCGTCCGTCATCAAGGGCGAGTCCCCCTCGACCCCGAAGTCCCTTATGTTGTGTCGGTTCTGCGTTTGTATTGCGGATACTATAGACCTGGACAGCCGTGGACCGCCCAGGATGCCACTATTCCGGCTGTTATCGTCAATGGTGATG 340

350 360 370 380 390 400 410 420 430 440 450 460 470 480 490 500 510

....|....|....|....|....|....|....|....|....|....|....|....|....|....|....|....|....|....|....|....|....|....|....|....|....|....|....|....|....|....|....|....|....|....|

**Tmel-MAT111**  AGGCCTTGGCCCGTATTCCCCCCTCTCAGCACCTTCGTGCATTGAATCCCTACGTTGCGCAGAGAT**GTAAGTAAATCACATCCCTATTTTGCCTCTATTTCTAACCATCTATTAG**CCTGGATTTCTAAGTACTGTAACGGTTATGGGCTCACTCAAGCCGAAATTTCCAA 510

**Ti_U986-MAT111** AGGCCTTGGCCCGCATCCCCCCCTCTCAGCACCTTCGTGCATTGAATCCCTACGTTGCGCAGAGAT**GTAAGTAAATCACATCCCTATTTTGCCTCTATTTCTAACCATCTATTAG**CCTGGATTTCTAAGTACTGTAACGGTTATGGGCTCACTCAAGCCGAAATTTCCAA 510

**Ti_U983-MAT111** AGGCCTTGGCCCGCATCCCCCCCTCTCAGCACCTTCGTGCATTGAATCCCTACGTTGCGCAGAGAT**GTAAGTAAATCACATCCCTATTTTGCCTCTATTTCTAACCATCTATTAG**CCTGGATTTCTAAGTACTGTAACGGTTATGGGCTCACTCAAGCCGAAATTTCCAA 510

**Ti_CF10-MAT111** AGGCCTTGGCCCGTATTCCCCCCTCTCAGCACCTTCGTGCATTGAATCCCTACGTTGCTCAGAGAT**GTAAGTAAATCACATCCCTATTTTGCCTCTATTTCTAACCATCTATTAG**CCTGGATTTCTAAGTACTGTAACGGTTATGGGCTCACTCAAGCCGAAATTTCCAA 510

520 530 540 550 560 570 580 590 600 610 620 630 640 650 660 670 680

....|....|....|....|....|....|....|....|....|....|....|....|....|....|....|....|....|....|....|....|....|....|....|....|....|....|....|....|....|....|....|....|....|....|

**Tmel-MAT111**  TCTCACTCGTGATGTCTGGGTCGCTGAGACCAATAAGTATATGTGGCAAAACATCGCCTCTCTTTATACTGCTGCTAGGGATCGTGGTGACCCCGGCCTCGTGCTCGAAGAATTTATCGAGACCGAATTGGCGAAACACGGTCACCCTACTACCCCTGAGAAGCTCTTGC 680

**Ti_U986-MAT111** TCTCACTCGTGATGTCTGGGTCGCTGAGACCAATAAGTATATGTGGCAAAACATTGCCTCTCTTTATACTGCTGCTAGGGATCGTGGTGACCCTGGTCTCGTGCTCGAAGAATTTATCGAGACCGAATTGGCGAAACACGGTCACCCTACTACCCCTGAGAAGCTCTTGC 680

**Ti_U983-MAT111** TCTCACTCGTGATGTCTGGGTCGCTGAGACCAATAAGTATATGTGGCAAAACATTGCCTCTCTTTATACTGCTGCTAGGGATCGTGGTGACCCTGGTCTCGTGCTCGAAGAATTTATCGAGACCGAATTGGCGAAACACGGTCACCCTACTACCCCTGAGAAGCTCTTGC 680

**Ti_CF10-MAT111** TCTCACTCGTGGCGTCTGGGTCGCTGAGACCAATAAGTATATGTGGCAAAACATCGCCTCTCTTTATACTGCTGCTAGGGATCGTGGTGACCCTGGTCTCGTGCTCGAAGAATTTATCGAGACCGAATTGGCGAAACATGGTCACCCTACTACCCCTGAGAAGCTCTTGC 680

690 700 710 720 730 740 750 760 770 780 790 800 810 820 830 840 850

....|....|....|....|....|....|....|....|....|....|....|....|....|....|....|....|....|....|....|....|....|....|....|....|....|....|....|....|....|....|....|....|....|....|

**Tmel-MAT111**  GTGAAGCTGGCTTTGTCCCCAAGGCCCCCATCGACAAGGAAGCGGCCGATAAGGCTGCTGCAAGAAGGAAGGTTCGTGCCAAGCCCGCACCCGGGGTGTCCAGTTTCACTATCACCCGTGTTTACGTTTCCAGCAACCGTCAGCTCGATCTCACCGAGGAGGAAGCCGCC 850

**Ti_U986-MAT111** GTGAAGCTGGCTTTGTCCCCAAGGCCCCCATCGACAAGGAAGCGGCCGATAAGGCTGCTGCAAGAAGGAAGGCTCGTGCCAAGCCCGCATCCGCGGTGTCCAGATTCACCATCACCCGTGTTTACGTTTCCAGCAACCATCAGCTCGATCTCACCGAGGAGGAAGCCGCC 850

**Ti_U983-MAT111** GTGAAGCTGGCTTTGTCCCCAAGGCCCCCATCGACAAGGAAGCGGCCGATAAGGCTGCTGCAAGAAGGAAGGCTCGTGCCAAGCCCGCATCCGCGGTGTCCAGATTCACCATCACCCGTGTTTACGTTTCCAGCAACCATCAGCTCGATCTCACCGAGGAGGAAGCCGCC 850

**Ti_CF10-MAT111** GTGAAGCTGGCTTTGTCCCCAAGGCCCCCATCGACAAGGAAGCGGCCGATAAGGCTGCTGCAAGAAGGAAGGCTCGTGCCAAGCCCGCACCCGCAGTGTCCAGATTCACTATCACCCGTGTTTACGTTTCCAGCAACCGTCAGCTCGATCTCACCGAGGAGGAAGCCGCC 850

860 870 880 890 900 910 920 930 940 950 960 970 980 990 1000 1010 1020

....|....|....|....|....|....|....|....|....|....|....|....|....|....|....|....|....|....|....|....|....|....|....|....|....|....|....|....|....|....|....|....|....|....|

**Tmel-MAT111**  AACATCCTCCATGCTTTCGACATCGCTAGCGCTGAGGAGAATGGAGACTCTCAACTCGACCCGCACCTCCAGCCCGAGATCGAGATGCACATGAACACTTTCACTTCTGGTGGATACAGCGTTTTT**GTGAGAAGTTCCCCGAACAGCATATTCCGCCACTGCCCAATTCT** 1020

**Ti_U986-MAT111** AACATCCTCAATGCTTTCGACATCGCTAGCGCTGAGGAGAATGGAGACTCTCAACTCGACCCGCACTTCCAGCCCGAGACCGAGATGCACATGAACACTTTCACTTCTGGTGGATACAGCGTTTTT**GTGAGAAGTTCCCCGAACAACATATTCCACCAGCGCCCAATTCT** 1020

**Ti_U983-MAT111** AACATCCTCAATGCTTTCGACATCGCTAGCGCTGAGGAGAATGGAGACTCTCAACTCGACCCGCACTTCCAGCCCGAGACCGAGATGCACATGAACACTTTCACTTCTGGTGGATACAGCGTTTTT**GTGAGAAGTTCCCCGAACAACATATTCCACCAGCGCCCAATTCT** 1020

**Ti_CF10-MAT111** AACATCCTCCATGCTTTTGACATCACTAGCGCTGAGGAGAATGGAGACTCTCAACTCGACCCGCACTTCCAGCCCGAGAACGAGATGCACATGAACACTTTCACTTCTGGTGGATACAGCGTTTTT**GTGAGAAGTTCCCCGAACAGCATATTCCGCCAGCCCCCAATTCT** 1020

1030 1040 1050 1060

....|....|....|....|....|....|....|....|....|....

**Tmel-MAT111**  **AACATTATCTAAACAG**CCTACTATTGGAGAATCCTCCACTGGATTGTGA 1069

**Ti_U986-MAT111 AACATGATCTAAACAG**CCTCCTATTGGAGAATCCTCCACTGGATTGTGA 1069

**Ti_U983-MAT111 AACATGATCTAAACAG**CCTCCTATTGGAGAATCCTCCACTGGATTGTGA 1069

**Ti_CF10-MAT111 AACATGATCTAAACAG**CCTACTATTGGAGAATCCTCCACTGGATTGTGA 1069
