# Supplementary material for: Mating Type Locus of Chinese Black Truffles Reveals Heterothallism and the Presence of Cryptic Species within the T. indicum Species Complex
Source: PLoS One. 2013 Dec 16;8(12):e82353. doi: 10.1371/journal.pone.0082353 (PMC3864998; doi:10.1371/journal.pone.0082353)
Supplement: Figure S7 — Nucleotide alignment of T. indicum and T. melanosporum MAT1-2-1 genes. Introns are shown in bold type. (DOC) [file pone.0082353.s007.doc]

**Figure S7 Nucleotide alignment of *T. indicum* and *T. melanosporum* *MAT1-2-1* genes.** Introns are shown in bold type underlined.

10 20 30 40 50 60 70 80 90 100 110 120 130 140 150 160 170

....|....|....|....|....|....|....|....|....|....|....|....|....|....|....|....|....|....|....|....|....|....|....|....|....|....|....|....|....|....|....|....|....|....|

**Tmel-MAT121**  ATGA---CTGCTTTGTCCATGCTCAATATGGCCGTTGAAGCCGGGCCTCTTATTCACCAACGTTATGATGGGATCGGTCTCGCCGAGTATATTCCCTTGCTCGCCAATGGAACTTTTTTCTTTGATGGGTCGGATGGAGCCCCGCTCCAGGTCCGTCATCTCCTTCCAGC 167

**Ti_CF10-MAT121** ATGACTTCTTCTTTGTCCATTCTCAATATGGCCGTTGAAGCCGGGCCTCTTATTCACCAACGTTATGATGGAATCGGTCTTGCCGAGTATATTCCCTTGCTCGCCAACGGAACCTTTTTCTTTGATGGGCCGGATGGAGTCTCCCTCCAAGTCCGCCATCTCCTTCCAGC 170

**Ti_U983-MAT121** ATGACTTCTTCTTTGTCCAACCTCAATATGGCCCTTGAAGCCGGGCCTCTTATTCACCAACGTTATGATGGAATCGGTCTCGCCGAGTATATTCCCTTGCTCGCCAACGGAACCTTTTTCTTTGATGGGTCGGATGGAGTCTCCCTCCAGGTCCGCCACCTCCTTCCAGC 170

**Ti_U986-MAT121** ATGACTTCTTCTTTGTCCAACCTCAATATGGCCCTTGAAGCCGGGCCTCTTATTCACCAACATTATGATGGAATCGGTCTCGCCGAGTATATTCCCTTGCTCGCCAACGGAACCTTTTTCTTTGATGGGTCGGATGGAGTCTCCCTCCAGGTCCGCCACCTCCTTCCAGC 170

180 190 200 210 220 230 240 250 260 270 280 290 300 310 320 330 340

....|....|....|....|....|....|....|....|....|....|....|....|....|....|....|....|....|....|....|....|....|....|....|....|....|....|....|....|....|....|....|....|....|....|

**Tmel-MAT121**  AGACTGGAAGCCCGTCAATGGACCACTTACGGTCTTGTTCGATTCCGCCCCCCATACCATTATTGGGACTCACTTCTCTATCCACAATCATCCCATTTACAAGCTTAACGATGGTTCTATCTTTGTTGTCTTGGACCTGGCCACCAAGCACGGTTTTGAGTTTACCAATC 337

**Ti_CF10-MAT121** AGACTGGAAGCCCGTCAATGGACCACTTACGGTCTTGTTCGATTCCGCCCCCCATACCATTATTGGGACTCATTTTTCTATCCGCAATCATCCCATTTATAAGCTTAACGATGGTTCTATCTTTGTCGTCTTGGACCCGGCCACCAAGCACGGTTTCGAGTTTACCGATC 340

**Ti_U983-MAT121** AGACTGGAAGCCCGTCAATGGACCACTTGCGGTCTTGTTCGATTCCGTCCCACATACCATTATTGGGACTCACTTTTCTATCCGCAATCACCCCATTTATAAGCTTAACGATGGTTCTATCTTGGTCGTCTTTGACCCGGCCGCTAAGCACGGTTTCGAGTTTACCAATC 340

**Ti_U986-MAT121** AGACTGGAAGCCCGTCAATGGACCACTTGCGGTCTTGTTCGATTCCGTCCCACATACCATTATTGGGACTCACTTTTCTATCCGCAATCACCCCATTTATAAGCTTAACGATGGTTCTATCTTGGTCGTCTTTGACCCGGCCGCTAAGCACGGTTTCGAGTTTACCAATC 340

350 360 370 380 390 400 410 420 430 440 450 460 470 480 490 500 510

....|....|....|....|....|....|....|....|....|....|....|....|....|....|....|....|....|....|....|....|....|....|....|....|....|....|....|....|....|....|....|....|....|....|

**Tmel-MAT121**  AATCTTTCATGGACCCCCAGCTTGAGGTCTCCGGGGAAAACCGTTTCTTGCGTCTCGTTACCAATGACCAAGACTTCGCCTACCATGGCTCTAGTCTTAGCATGATTCATGACAAGGGCGTTAAGCGCCCTGCCAATGC**GTA--AGTACAATTCTACCAATGTACCAGTT** 505

**Ti_CF10-MAT121** AATCTTTCGTGGACCCCCAGCTTGAGGTCTCCGGAGAAAACCGTTTCTTGCGTCTCGTTACCAATGACCAAGACTTCGCCTACCATGGTTCTAGTCTTAGCATGATTCATGACAAGGGCGTTAAGCGCCCTGCCAATGC**GTATTGATACAATTCTACCAATGTACCAGTT** 510

**Ti_U983-MAT121** AATCTTTCGTGGACCCCCAGCTTGAGGTCTCCGGAGAGAACCGTTTCTTGCGTCTCGTTACCAATGACCAAGACTTCGCCTACCATGGTTCTAGTCTTAGCATGATTCATGACAAGGGCGTTAAGCGCCCTGCCAATGC**GTA--AGTACAATTCTACCAATGTACCAGTT** 508

**Ti_U986-MAT121** AATCTTTCGTGGACCCCCAGCTTGAGGTCTCCGGAGAGAACCGTTTCTTGCGTCTCGTTACCAATGACCAAGACTTCGCCTACCATGGTTCTAGTCTTAGCATGATTCATGACAAGGGCGTTAAGCGCCCTGCCAATGC**GTA--AGTACAATTCTACCAATGTACCAGTT** 508

520 530 540 550 560 570 580 590 600 610 620 630 640 650 660 670 680

....|....|....|....|....|....|....|....|....|....|....|....|....|....|....|....|....|....|....|....|....|....|....|....|....|....|....|....|....|....|....|....|....|....|

**Tmel-MAT121**  **TCGTGTGCTCATATAGTCATAG**GTTTATCTTGTTCAGAACGGACAAAATGAAGCCCCTCAAGGCTCAGTACCCGGAGATGGGCAACAACGATATCT**GTACGTGGCTCTCATTTTCCCTTTATTTAGATCTCTATTCTTGCTACCATTTGCCTAACGTTTTAATAG**CCAAG 675

**Ti_CF10-MAT121 TCGTGTGCTCATCTAGTCATAG**GTTCATTTTATTCAGAACGGACAAAATGAAGCCCCTCAAGGCTCAGTACCCGGAGATGGGCAACAACGATATCT**GTACGTGGCTCTCATTTCCCCCTTATTTAGATCTCTATTCTTGGTACCATTTGCCTAACGTTTTAATAG**CCAAG 680

**Ti_U983-MAT121 TCGTGTGCTCATCTAGTCATAG**GTTTATCTTATTCAGAACGGACAAAATGAAGCCCCTCAAGGCTCAGTACCCGGAGATGGGCAACAACGATATCT**GTATGTGGCTCTTATTTTCCCTTTATTTTGATCTCTATTCTTGGTACCATTTGCCTAACGTTTTAATAG**CCAAG 678

**Ti_U986-MAT121 TCGTGTGCTCATCTAGTCATAG**GTTTATCTTATTCAGAACGGACAAAATGAAGCCCCTCAAGGCTCAGTACCCGGAGATGGGCAACAACGATATCT**GTATGTGGCTCTTATTTTCCCTTTATTTTGATCTCTATTCTTGGTACCATTTGCCTAACGTTTTAATAG**CCAAG 678

690 700 710 720 730 740 750 760 770 780 790 800 810 820 830 840 850

....|....|....|....|....|....|....|....|....|....|....|....|....|....|....|....|....|....|....|....|....|....|....|....|....|....|....|....|....|....|....|....|....|....|

**Tmel-MAT121**  ATTCTCGGTCGCATGTGGCAGAATTCCTCGGATGAGGTCAAAAATGTTTATAAG**GTAAAACATCTCACCTAACTGTTAGTTAGAATCACTAACACATTAATAG**GCAAGGGCCCATCAGCTTGCGGTTTCCCACAAGTTGATCAACCCGGATTACAAATACTCCCCGCGCC 845

**Ti_CF10-MAT121** ATTCTCGGTCGCATGTGGCAGAATTCCTCGGATGAGGTCAAAGATGTTTATAAG**GTAAAACATCTCACCTAACTGTTAGTTAGAATCACTAACACATTAATAG**GCAAGGGCCCATCAGCTTGCGGTTTCCCATAAGTTGATCAACCCGGATTACAAATACTCCCCGCGCC 850

**Ti_U983-MAT121** ATTCTCGGTCGCATGTGGCAGAATTCCTCGGATGAGGTCAAAGATGTTTATAAG**GTAAAACATCTCACCTAACTGTTAGTTAGAATCACTAACACATTAATAG**GCGAGGGCCCATCAGCTTGCGGTTTCCCATAAGTTGACCAACCCGGATTACAAATACTCCCCGCGCC 848

**Ti_U986-MAT121** ATTCTCGGTCGCATGTGGCAGAATTCCTCGGATGAGGTCAAAGATGTTTATAAG**GTAAAACATCTCACCTAACTGTTAGTTAGAATCACTAACACATTAATAG**GCGAGGGCCCATCAGCTTGCGGTTTCCCATAAGTTGACCAACCCGGATTACAAATACTCCCCGCGCC 848

860 870 880 890 900 910 920 930 940 950 960 970 980 990 1000 1010 1020

....|....|....|....|....|....|....|....|....|....|....|....|....|....|....|....|....|....|....|....|....|....|....|....|....|....|....|....|....|....|....|....|....|....|

**Tmel-MAT121**  GCTCTCGCGAAATCATGAGGAGGGGCTCCGGGAATAAAGGTCCTTCGGCCCGTATCGACCTTCATCGTGCTGGTGTTGCGAAGCGTGGAAAAAAGAGGATCATTACCCACCTTGGGTTCCTCACTCAACGTTTCGCGACTCACCCCACTGCTAACAATCTCGGTGATATC 1015

**Ti_CF10-MAT121** GCTCTCGCGAAATCATGAGGAGGGACTCCGGGAATAAAGGTCCTTCCCCCCGTATCGACCTTCACCGTGCTGGCGTTGCGAAGCGTGGAAAAAAGAGGATCGTTACCCACCTTGGGTTCCTCACTCAACGTTTCGCGACTCACCCCACTGTTAACAATCTCGGTGATATC 1020

**Ti_U983-MAT121** GCTCTCGCGAAATCATGAGGAGGGACTCCGGGAATAAAGGTCCTTCTCCCCGTATCGACCTTCATCGTGCTGGTGTTGCGAAGCGTGAAAAAAAGAGGATCATTACCCACCTTGGGTTCCTCACTCAACGTTTCGCAACTCACCCCACTGCTAACAATCTCGGTGATATC 1018

**Ti_U986-MAT121** GCTCTCGCGAAATCATGAGGAGGGACTCCGGGAATAAAGGTCCTTCTCCCCGTATCGACCTTCATCGTGCTGGTGTTGCGAAGCGTGAAAAAAAGAGGATCATTACCCACCTTGGGTTCCTCACTCAACGTTTCGCAACTCACCCCACTGCTAACAATCTCGGTGATATC 1018

1030 1040 1050 1060

....|....|....|....|....|....|....|....|....|..

**Tmel-MAT121**  ATCGAGGAAGCCGAAAAGGTCTTGAAGGCCTTTGGTATGGTCAATTG 1062

**Ti_CF10-MAT121** ATCGAGGAAGCCGAAAAGGTCTTGAAGGCCTTTGGTATGGTCAATTG 1067

**Ti_U983-MAT121** ATCGAGGAAGCCGAAAAGGTCTTGAAGGCCTTTGGTATGGTCAATTG 1065

**Ti_U986-MAT121** ATCGAGGAAGCCGAAAAGGTCTTGAAGGCCTTTGGTATGGTCAATTG 1065
