# Supplementary material for: Mating Type Locus of Chinese Black Truffles Reveals Heterothallism and the Presence of Cryptic Species within the T. indicum Species Complex
Source: PLoS One. 2013 Dec 16;8(12):e82353. doi: 10.1371/journal.pone.0082353 (PMC3864998; doi:10.1371/journal.pone.0082353)

**Figure S8 Alignment of *Trp1* encoded hypothetical protein with Piggybac transposases**. Black arrows indicate the conserved DDD catalytic triad; red arrows indicate conserved amino acidic residues as reported by Youan and Wessler [47]. The default ClustalX color code is used.


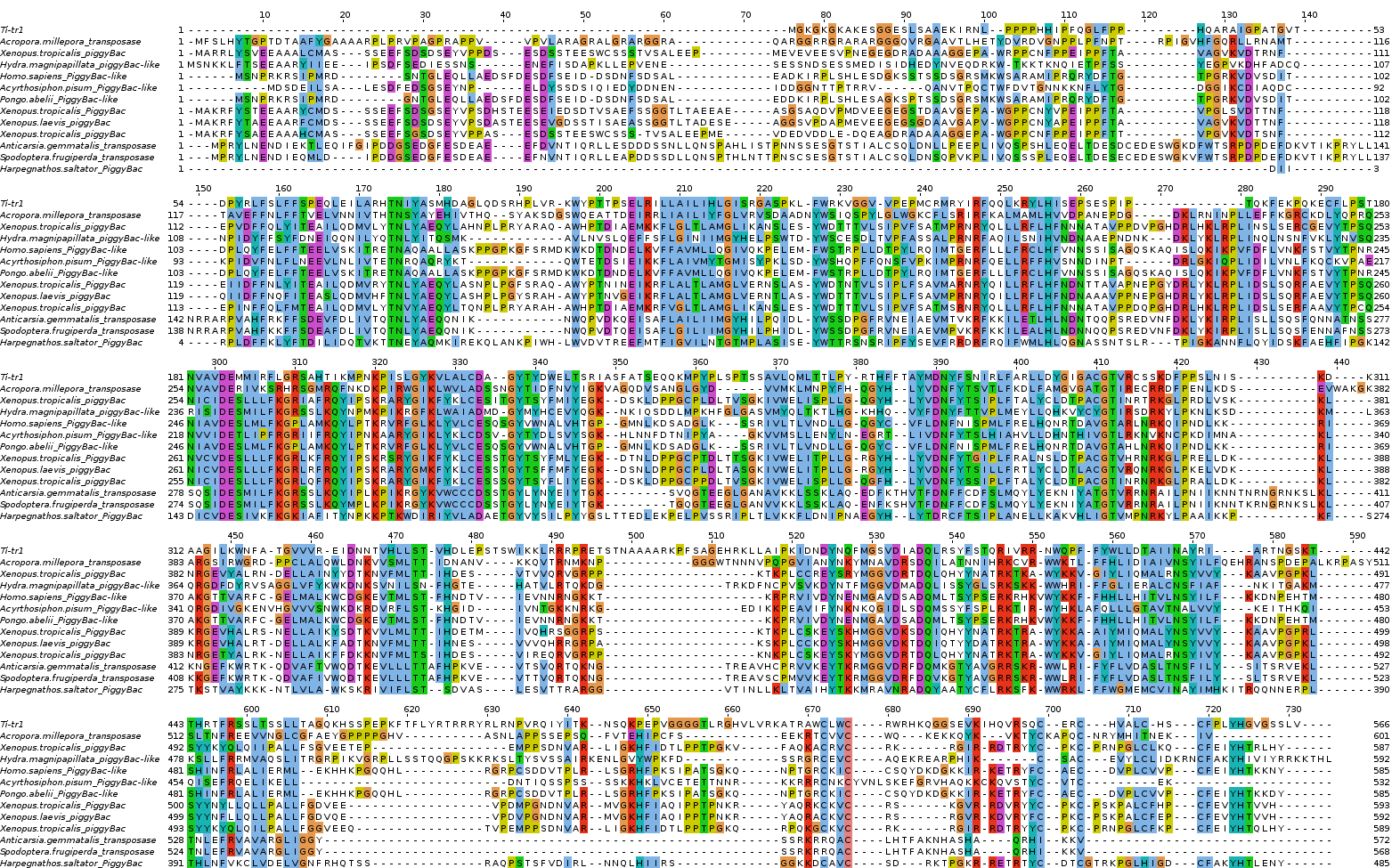

Supplement: Figure S8 — Alignment of Trp1 encoded hypothetical protein with Piggybac transposases. Black arrows indicate the conserved DDD catalytic triad; red arrows indicate conserved amino acid residues as reported by Youan and Wessler [47]. The default ClustalX color code is used. (DOC) [file pone.0082353.s008.doc]
