# Supplementary material for: Mating Type Locus of Chinese Black Truffles Reveals Heterothallism and the Presence of Cryptic Species within the T. indicum Species Complex
Source: PLoS One. 2013 Dec 16;8(12):e82353. doi: 10.1371/journal.pone.0082353 (PMC3864998; doi:10.1371/journal.pone.0082353)
Supplement: Figure S9 — Alignment of Trp2 encoded hypothetical protein with TC1/mariner transposases. Black arrows indicate the conserved DD(D/E) catalytic triad; red arrows indicate conserved amino acid residues as reported by Youan and Wessler [47]. The default ClustalX color code is used. (DOC) [file pone.0082353.s009.doc]

**Figure S9 Alignment of *Trp2* encoded hypothetical protein with TC1/mariner transposases**. Black arrows indicate the conserved DD(D/E) catalytic triad; red arrows indicate conserved amino acidic residues as reported by Youan and Wessler [47]. The default ClustalX color code is used.

**
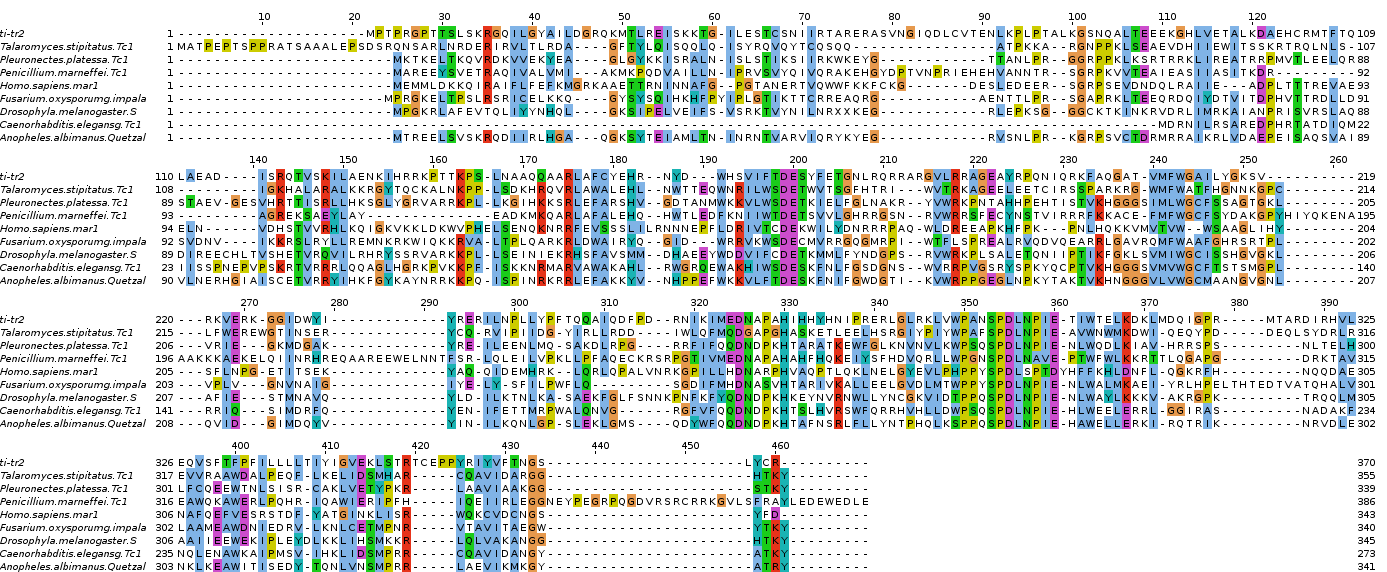
**
