# Supplementary material for: Mating Type Locus of Chinese Black Truffles Reveals Heterothallism and the Presence of Cryptic Species within the T. indicum Species Complex
Source: PLoS One. 2013 Dec 16;8(12):e82353. doi: 10.1371/journal.pone.0082353 (PMC3864998; doi:10.1371/journal.pone.0082353)
Supplement: Figure S10 — Nucleotide sequence and structure of Ti_tr1 transposon. A) Nucleotide alignment of Ti_tr1 TIR. B) Organization of the Ti_tr1 transposon. TIRs are indicated in Bold; the TSDs in red; the putative transposase CDS in blue; the putative start and stop codons are underlined. (DOC) [file pone.0082353.s010.doc]

**Figure S10 Nucleotide sequence and structure of Ti_tr1 transposon.** A) Nucleotide alignment of Ti_tr1 TIR. B) Organization of the Ti_tr1 transposon. TIRs are indicated in Bold; the TSDs in red; the putative transposase CDS in blue; the putative start and stop codons are underlined.

**A)**

10 20 30 40 50
Ti-tr1(5'TIR) : TCCCTTAGAGAGCTGAATCAACGAAATTCAGTGCCGCCAAGATATTGCGCAA : 52
Ti-tr1(3'TIR) : TCCCTTCGAGAGCTGAAACAACGAAATTCAGTGCCGCCAAGATATTGCGCAA : 52

**B)**

//ttgtggctgttgcatcgaactaccatatgactttcctaaataggaaggcagactcttg

attcttttagatggcctggcagttgttgaaggttctgaac**tatcccttagagagctgaat**

**caacgaaattcagtgccgccaagatattgcgcaa**ctgtgctgtctaagacatagggtgaa

taagcactaagcgacgtcgtgagaagctgaatctcgtgagaagattaagaaaacaaatcc

caatcgttcagaattctactagaaatgccacgaacaagaaaatcaagtgcacaaacggct

gctagaggagtggtgtcccagtcggttcaccagagagagcctttagctgtcctatcagtg

aacggtaggaagccaagccgaatatgttcatagaatgctaaacgtttcacagcaggtgca

gaatctcgaatgggaccagtggggccatcacatctgcatatggataccataatgcctatg

ggtgag**atgggaaagggaaagggaaaagcaaaggagtcgggtggggagtcactctctgcg**

**gccgaaaagattcgaaatcttcctcctccgccacaccacataccattccaaggactattt**

**ccaccgcaccaagctagagccataggacctgcaactggagtaactgacccgtatcggtta**

**ttctcactgtttttttcgccggagcaactggagattctggcgaggcataccaatatttat**

**gcaagtatgcatgatgctggcctgcaagacagtaggcaccctcttgttcggaagtggtat**

**ccaacaactccctctgagcttcgaatattgttagcgatcttgattcatctgggtatatca**

**agaggtgctagtccaaaactcttttggagaaaggttggaggtgttgttccggaaccgatg**

**tgcagaatgagatatatacgctttcagcagctaaagcgctatttgcatatctcagagcca**

**tctgagtccccaattcctactcaaaa**gtggtggaaaaagctcgaacctttaaatagcag**t**

**attcgaaaaacctcaaaaagagtgtttccttccgtccaccaatgtggctgtcgatgagat**

**gatgatacgcttccttggccgctcagcccacacaattaaaatgccaaataaacctattag**

**tttaggatataaggtactggcattgtgtgatgcaggatacacctatgactgggaacttac**

**ttcacgtattgcgagctttgcaacctccgaacagcaaaaaatgccatatcctctttctcc**

**tacctcctccgctgtacttcagatgcttaccacactaccctaccgtacccacttttttac**

**cgcctatatggacaattacttctcaaatatacgcctatttgcacgtttactggattatgg**

**aattggagcctgtggtacagtgcggtgcagcagcaaagattttcctccctctctcaatat**

**cagcaaagataaggctgctggtatcttgaaatggaattttgctactggagttgttgtacg**

**cgaaata**gttgatacagcaaaaaagataaagggagacagcccacccaagctacataggtg

ggggattcctccggttatggcttttttgtggcag**gataataacacggtgcatttattatc**

**tactgttcatgatcttgagccatcaacttcctggataaagaaacttcgcagaaggccacg**

**ggaaacaagtaccaatgctgctgctgctcggaagcccttttctgctggggagcatcgcaa**

**gctattggcaattcccaagatcgacaatgactataaccaattcatgggtagtgtggatat**

**tgcagatcagcttcgatcatatttttctacgcaacgtattgtgcgtcggaactggcaacc**

**ttttttttactggttacttgatactgcaatcattaatgcctaccgaatcgcgcgaaccaa**

**tggctctaaaaccacccaccgtacatttcgctcctcccttacaagttcccttcttactgc**

**cggtcagaaacactcctcccctgagccaaaatttacattcctctaccgaactcgacgacg**

**gtatcgcctacgcaacccagtacgtcaaatatatattacgaagaactcacagaagccaga**

**accggtaggtggagggggaactttaaggggccatgtactggtacggaaagctactcgagc**

**atggtgcctatggtgccgctggcgtcacaagcaaggtggctcggaggtgaaaatccacca**

**agtgcgatctcagtgtgaacggtgtcatgtagccctttgtcattcatgttttcccttata**

**tcatggtgtaggaagctctttggta**taggaagctcttcggtctaggttctcttttttttt

tttttttatatcactttgtctatttagtaatccagatccctgctctggcttgctgtcga**t**

**tgcgcaatatcttggcggcactgaatttcgttgtttcagctctcgaagggata**ggaacta

ttggtacttgtactaaatactaattcctaacaacgtacagggatgtcacgcaaaatcaaa

tcaaacattacacactaatggtattaaa//
