# Supplementary material for: Mating Type Locus of Chinese Black Truffles Reveals Heterothallism and the Presence of Cryptic Species within the T. indicum Species Complex
Source: PLoS One. 2013 Dec 16;8(12):e82353. doi: 10.1371/journal.pone.0082353 (PMC3864998; doi:10.1371/journal.pone.0082353)
Supplement: Figure S11 — Nucleotide sequence and structure of Ti_tr2 transposon. A) Nucleotide alignment of Ti_tr2 TIR. B) Organization of the Ti_tr2 transposon. TIRs are indicated in Bold; the TSDs in red; the putative transposase CDS in blue; the putative start and stop codons are underlined. (DOC) [file pone.0082353.s011.doc]

**Figure S11 Nucleotide sequence and structure of Ti_tr2 transposon.** A) Nucleotide alignment of Ti_tr2 TIR. B) Organization of the Ti_tr2 transposon. TIRs are indicated in Bold; the TSDs in red; the putative transposase CDS in blue; the putative start and stop codons are underlined.

**A)**

10 20 30 40
Ti-tr2(5'TIR) : CTCGTAATGTCGTGACGTTTGTGAACACGACCGGGCTTGCATTAG : 45
Ti-tr2(3'TIR) : CTCGTAATGTCGTGACGTTTGTGAACACGACCGGGCTTGCATTAG : 45

**B)**

gttggttctcatgaactagttttcgcaaccctgttaacctgtcagacgtatgatagcatg

tcgtgcgctgatgcggcgaccaacagggatattggagacctacggagatatagaatcgaa

aggatttttccagtattgg**tactcgtaatgtcgtgacgtttgtgaacacgaccgggcttg**

**cattagtgtacattag**tgtgaatacaagggggtaacagacgagaaatgaacttaatgtag

actagttagtaaataggggtacttcagtgaataggggtacttgagtatatgaagttcgtg

atcaatagtagcaatacagtgaatccaatatccagcaaaa**atgccaactccacgaggtcc**

**tacaacttctttgagcaagcgaggtcagattttgggttatgcaattctcgatgggcgaca**

**gaaaatgacattgagggagatatcgaaaaagacaggaatacttgaaagtacttgttcgaa**

**tattatccgaactgcacgcgagcgagcgagtgtgaatggcatccaagatctctgcgtaac**

**tgagaatctaaagccattgcctaccgcactcaaaggatcaaatcaggctcttaccgagga**

**agaaaagggtcatcttgttgaaacagcactgaaggatgctgagcactgtcgaatgacctt**

**tacgcagcttgctgaagctg**gtatgtattttatattaaaattccctataatacataaatg

taactaaaaaaaaaaaaaaaaaaagccaatttag**atatcagtcggcagactgtatctaag**

**atactagctgaaaataaaatccatcgccgaaagccaacaacaaaaccctcactcaatgca**

**gctcaacaggcagcccggcttgccttttgctatgagcatcggaactacgattggcactct**

**gtaatttttacagacgagtcttattttgaaactggcaaccttcgccagcgccgtgcccgt**

**ggtgttttacgccgggctggtgaggcctaccggcctcaaaacattcagcgaaagtttgct**

**caaggagcaacggtcatgttttggggggctattttgtatggtaaatcaggtataatttat**

**ttattattattattgttattgtttattactatttctctttctcttactatttttattatt**

**tttaat**gtacatataggctaatataatatattaaaaaaggaaatgaactaccatatcatt

tatatgcttcaccatatgaaacagcgggacag**cggagagagtcatcaattatcctacagc**

**gagaatacgagttcgagttagcagaaaatactcgcgctactgtacttggacatgtagtcc**

**catttccaacacctgaattaaaggtgcggaaggtagagagaaagggagggatagattggt**

**atatctatcgtgaaagaattttaaatccccttctttaccctttcacccagcaagcaatac**

**aggactttcctgatcggaatattaaaataatggaggataatgcgcctgcgcatatccacc**

**attatcataatattccgcgagagcgtttggggctacggaagctagtctggccggccaact**

**cccccgacttgaatcccatagagactatatggactgagcttaaagataagctgatggatc**

**aaattggtccacggatgactgctcgtgatatccgccatgtacttgaacaagtaagcttta**

**ccttcccttttattcttttactactaacaatttacataggagtggagaaactatccacga**

**gaacgtgtgaaccaccatatagaatctatgtcttcacgaatggaagcctgtattgcagat**

**aa**tggtggaaacaactttaatttttaatattaaattttgtatttctttctttctcttttg

attccatgagtgctgtattcaccatatataattcgtaatatattatcatacctttatatc

cccctccctagtttcgccaagcataagcctcatatgcacg**ctaatgcaagcccggtcgtg**

**ttcacaaacgtcacgacattacgagta**gatggctgttagactcccttgcctccctccttt

tgctacgcacgatgaagaatagaacaatatcggtaggtta
