# Supplementary material for: Mating Type Locus of Chinese Black Truffles Reveals Heterothallism and the Presence of Cryptic Species within the T. indicum Species Complex
Source: PLoS One. 2013 Dec 16;8(12):e82353. doi: 10.1371/journal.pone.0082353 (PMC3864998; doi:10.1371/journal.pone.0082353)
Supplement: Figure S12 — Nucleotide alignment of Ti_tr1 and Ti_tr2 TIRs. (DOC) [file pone.0082353.s012.doc]

**Figure S12 Nucleotide alignment of Ti_tr1 and Ti_tr2 TIRs.**

10 20 30 40 50
Ti-tr2(5'TIR) : ---CTCGTAATGTCG---TGACGTTTGTGAACACGACCGGG--CTTGCATTAG : 45
Ti-tr2(3'TIR) : ---CTCGTAATGTCG---TGACGTTTGTGAACACGACCGGG--CTTGCATTAG : 45
Ti-tr1(5'TIR) : TCCCTTAGAGAGCTGAATCAACGAAATTCAGTGCCGCCAAGATATTGCGCAA- : 52
Ti-tr1(3'TIR) : TCCCTTCGAGAGCTGAAACAACGAAATTCAGTGCCGCCAAGATATTGCGCAA- : 52
