# Supplementary material for: Mating Type Locus of Chinese Black Truffles Reveals Heterothallism and the Presence of Cryptic Species within the T. indicum Species Complex
Source: PLoS One. 2013 Dec 16;8(12):e82353. doi: 10.1371/journal.pone.0082353 (PMC3864998; doi:10.1371/journal.pone.0082353)
Supplement: Figure S13 — Phylogenetic tree showing the relationship of T. indicum Trp1 and Trp2 encoded transposases with those of others related TEs. Phylogenetic tree was inferred with the Neighbor-Joining method and the Poisson distance model using the software Mega v. 5.05. Numbers near the branches indicate the bootstrap values (percentage of 1000 replicates). (DOC) [file pone.0082353.s013.doc]

**Figure S13 Phylogenetic tree showing the relationship of *T. indicum Trp1* and *Trp2* encoded transposases with those of others related TEs.** Phylogenetic tree was inferred with the Neighbor-Joining method and the Poisson distance model using the software Mega v. 5.05. Numbers near the branches indicate the bootstrap values (percentage of 1000 replicates).

*Homo.sapiens* (NP689808)

*Pongo.abelii* (XP002825300)

*Harpegnathos.saltator* (EFN83686)

*Hydra.magnipapillata* (XP 002155850)

*Danio rerio* (XP002667755)

*Talaromyces marneffei* (XP 002144531)

*Drosophyla.melanogaster* (AAC47095)

*Fusarium.oxysporum* (AAB33090)

*Talaromyces.stipitatus* (XP 002485610)

*Penicillium.marneffei* (XP 002146479)

*Xenopus.tropicalis* (XP002937731)

*Xenopus.tropicalis* (BAF82022)

*Xenopus.tropicalis* (BAF82019)

*Xenopus.laevis* (BAF82020.1)

*Acyrthosiphon.pisum* (XP001951956)

*Anticarsia.gemmatalis* (ACR78131)

*Spodoptera.frugiperda* (ACV83458)

*Acropora.millepora* (ACT79639)

**Trp1**

*Mucor circinelloydes* (Repbase)

*Homo.sapiens* (AAC52010)

*Anopheles.albimanus* (AAB02109)

*Caenorhabditis.elegans* (CAA25498)

*Pleuronectes platessa* (CAC28060)

**Trp2**

100

100

62

71

86

38

36

44

99

100

100

100

99

100

95

75

80

100

23

56

37

28

0.2

PiggyBac/PiggyBac like

Tc1/Mariner
