# Supplementary material for: Mating Type Locus of Chinese Black Truffles Reveals Heterothallism and the Presence of Cryptic Species within the T. indicum Species Complex
Source: PLoS One. 2013 Dec 16;8(12):e82353. doi: 10.1371/journal.pone.0082353 (PMC3864998; doi:10.1371/journal.pone.0082353)
Supplement: Table S1 — T. indicum samples analyzed. A, B1, B2 indicates the three ITS/RFLP patterns obtained with the Rsa1 endonuclease. (DOC) [file pone.0082353.s014.doc]

**Table S1 *T. indicum* samples analyzed. A, B1, B2 indicates the three ITS/RFLP patterns obtained with the *Rsa*1 endonulease.**

| Sample | RFLP pattern | Sample | RFLP pattern | Sample | RFLP pattern | Sample | RFLP pattern |
| --- | --- | --- | --- | --- | --- | --- | --- |
| Ti_C1^a^ | B1 | Ti_C67^a^ | A | Ti_D39^c^ | B1 | Ti_AR1^d^ | B1 |
| Ti_C2 ^a^ | B2 | Ti_C69^a^ | A | Ti_D40^c^ | B1 | Ti_AR2^d^ | A |
| Ti_C3 ^a^ | B2 | Ti_C70^a^ | A | Ti_D41^c^ | B1 | Ti_AR3^d^ | B1 |
| Ti_C4 ^a^ | A | Ti_C80^a^ | A | Ti_D43^c^ | B1 | Ti_AR4^d^ | A |
| Ti_C7 ^a^ | B2 | Ti_C81^a^ | A | Ti_D44^c^ | B1 | Ti_AR5^d^ | B1 |
| Ti_C8 ^a^ | B2 | Ti_CU3^b^ | A | Ti_D46^c^ | B1 | Ti_U981^d^ | B1 |
| Ti_C9 ^a^ | B2 | Ti_CU20^b^ | B1 | Ti_D49^c^ | B1 | Ti_U982^d^ | B1 |
| Ti_C14 ^a^ | B1 | Ti_CU27^b^ | B1 | Ti_D50^c^ | B1 | Ti_U983^d^ | B1 |
| Ti_C15 ^a^ | B2 | Ti_F2^c^ | B1 | Ti_D51^c^ | B1 | Ti_U984^d^ | B1 |
| Ti_C18 ^a^ | B2 | Ti_F3^c^ | B1 | Ti_D52^c^ | B1 | Ti_U985^d^ | B1 |
| Ti_C20 ^a^ | B1 | Ti_F6^c^ | B1 | Ti_D53^c^ | B1 | Ti_U986^d^ | B2 |
| Ti_C21 ^a^ | A | Ti_F7^c^ | B1 | Ti_D54^c^ | B1 | Ti_U987^d^ | B1 |
| Ti_C22 ^a^ | B1 | Ti_F9^c^ | B1 | Ti_D55^c^ | B1 | Ti_U988^d^ | B1 |
| Ti_C23 ^a^ | B1 | Ti_D1^c^ | B1 | Ti_D56^c^ | A | Ti_U989^d^ | B1 |
| Ti_C24 ^a^ | A | Ti_D3^c^ | A | Ti_RIBA^c^ | B1 | Ti_U9810^d^ | B1 |
| Ti_C27 ^a^ | B2 | Ti_D5^c^ | A | Ti_RIBB^c^ | B1 | Ti_U9811^d^ | B1 |
| Ti_C29 ^a^ | B2 | Ti_D6^c^ | B1 | Ti_RIBC^c^ | B1 | Ti_U9812^d^ | B1 |
| Ti_C30 ^a^ | B1 | Ti_D9^c^ | A | Ti_P1^c^ | B1 | Ti_U9813^d^ | B1 |
| Ti_C31 ^a^ | B2 | Ti_D13^c^ | B1 | Ti_P2^c^ | B1 | Ti_U9814^d^ | B1 |
| Ti_C34 ^a^ | B1 | Ti_D15^c^ | B1 | Ti_P3^c^ | B1 | Ti_CF2^e^ | B2 |
| Ti_C36 ^a^ | B1 | Ti_D23^c^ | B1 | Ti_P4^c^ | B1 | Ti_CF3^e^ | A |
| Ti_C37 ^a^ | A | Ti_D26^c^ | B1 | Ti_LI1^c^ | B1 | Ti_CF4^e^ | A |
| Ti_C38 ^a^ | B2 | Ti_D28^c^ | B1 | Ti_LI2^c^ | B1 | Ti_CF5^e^ | A |
| Ti_C40 ^a^ | B1 | Ti_D31^c^ | B1 | Ti_LI3^c^ | B1 | Ti_CF6^e^ | A |
| Ti_C47 ^a^ | A | Ti_D34^c^ | B1 | Ti_LI4^c^ | B1 | Ti_CF7^e^ | B2 |
| Ti_C55 ^a^ | A | Ti_D35^c^ | B1 | Ti_LI5^c^ | B1 | Ti_CF10^e^ | A |
| Ti_C57 ^a^ | A | Ti_D36^c^ | B1 | Ti_LI6^c^ | B1 | Ti_CF11^e^ | A |
| Ti_C61 ^a^ | B2 | Ti_D37^c^ | B1 | Ti_LI7^c^ | B1 | Ti_CF14^e^ | B1 |
| Ti_C66 ^a^ | A | Ti_D38^c^ | B1 | Ti_LI8^c^ | B2 |  |  |

Truffles imported in Italy in 1995(^a^), 1996 (^b^), 1997 (^c^) and 1998(^d^); Truffles imported in France in 1996 (^e^).
