# Supplementary material for: Mating Type Locus of Chinese Black Truffles Reveals Heterothallism and the Presence of Cryptic Species within the T. indicum Species Complex
Source: PLoS One. 2013 Dec 16;8(12):e82353. doi: 10.1371/journal.pone.0082353 (PMC3864998; doi:10.1371/journal.pone.0082353)
Supplement: Table S2 — List of primers used in this study. (DOC) [file pone.0082353.s015.doc]

**Table S2 List of primers used in this study**

| Primer name | Primer sequence |
| --- | --- |
| i1 | ACGTGGCAYTCTCTATTGGAGGATTCTG |
| i2 | ATYTTGCGCAGTTTATKGTCGCTCACAG |
| i3 | CCAGAAGTGAAAGTGTTCATGTGCATCTCG |
| i4 | CCCTACTACCCCTGAGAAGCTCTTGCG |
| i5 | ATGGACCACTTRCGGTCTTGTTCGATTC |
| i6 | TGGTAGGCGAAGTCTTGGTCATTGGTAAC |
| i7 | TCCTGCCTTAGGCCAATCCGCTTT |
| i8 | GGTTCCAGGCAGTGGCTTCCATTTCA |
| i9 | TGGACATGGGATCGGGTGGGAATAC |
| i10 | GGGATTGGGTGGGAGTATTGATCCTCG |
| i11 | TATCGGCTCACAATCCAGTGGAGGATTC |
| i12 | ACGGCCCAGGATGCCACTATTC |
| i13 | CCGTTGAGATCACTCAATTGACCATACC |
