# Supplementary material for: Mating Type Locus of Chinese Black Truffles Reveals Heterothallism and the Presence of Cryptic Species within the T. indicum Species Complex
Source: PLoS One. 2013 Dec 16;8(12):e82353. doi: 10.1371/journal.pone.0082353 (PMC3864998; doi:10.1371/journal.pone.0082353)
Supplement: Table S3 — Nucleotide differences between T. indicum and T. melanosporum MAT1-2-1 and MAT1-1-1 genes. (DOC) [file pone.0082353.s016.doc]

**Table S3 Nucleotide differences between *T. indicum* and *T. melanosporum* *MAT1-2-1* and *MAT1-1-1* genes**

| **MAT1-1-1** | | | | |
| --- | --- | --- | --- | --- |
|  | *T. melanosporum* | Ti_CF10 | Ti_U983 | Ti_U986 |
| *T. melanosporum* |  |  |  |  |
| Ti_CF10 | 27^*^ |  |  |  |
| Ti_U983 | 26^*^ | 28^*^ |  |  |
| Ti_U986 | 28^*^ | 30^*^ | 2^*^ | 0 |
|  |  |  |  |  |
| ***MAT1-2-1*** | | | | |
|  | *T. melanosporum* | Ti_CF10 | Ti_U983 | Ti_U986 |
| *T. melanosporum* |  |  |  |  |
| Ti_CF10 | 46 (41^*^ + 5^**^) |  |  |  |
| Ti_U983 | 48 (45^*^ + 3^**^) | 38 (36^*^ + 2^**^) |  |  |
| Ti_U986 | 49 (46^*^ + 3^**^) | 39 (37^*^ + 2^**^) | 1^*^ | 0 |
|  |  |  |  |  |
| ***MAT1-1-1 + MAT1-2-1*** | | | | |
|  | *T. melanosporum* | Ti_CF10 | Ti_U983 | Ti_U986 |
| *T. melanosporum* |  |  |  |  |
| Ti_CF10 | 73 (68^*^ + 5^**^) |  |  |  |
| Ti_U983 | 74 (71^*^ + 3^**^) | 66 (64^*^ + 2^**^) |  |  |
| **Ti_U986** | 77 (74^*^ + 3^**^) | 69 (67^*^ + 2^**^) | 3^*^ | 0 |

^*^ = substitutions; ^**^ = indels
